# The multi-pathway treatment of flavonoids as natural compounds in neurological diseases: achievements, limitations, and prospects

**DOI:** 10.3389/fnins.2025.1690170

**Published:** 2025-11-05

**Authors:** Yuzhu Fan, Xiaotong Lian, Xudong Ren, Liqun Ren, Cuizhe Liu, Yanbin Meng, Lin Zhang

**Affiliations:** Hebei Key Laboratory of Nerve Injury and Repair, Hebei Province Key Laboratory of Research and Development for Chinese Medicine, Institute of Traditional Chinese Medicine, Chengde Medical University, Chengde, Hebei, China

**Keywords:** flavonoids, neurological diseases, neuroprotection, anti-inflammation, anti-oxidation

## Abstract

**Background:**

The nervous system governs fundamental life activities and higher neural functions, maintaining the body’s interaction with the external environment and internal homeostasis. Neurological diseases are a common and complex group of disorders that severely impair patients’ quality of life and physical health. Flavonoids, as a class of active components widely present in natural plants, play a crucial role in the research on neurological diseases.

**Objective:**

The purpose of this review is to systematically sum up the action mechanisms, research advancements, and existing clinical uses of flavonoids in preventing and treating neurological diseases, probing into their potential in the management of neurological diseases.

**Methods:**

By searching databases including PubMed, Web of Science, Embase, Wiley, Springer, NPG, ACS, Elsevier, and RSC, literature published between 2000 and 2025 concerning the treatment of neurological diseases with flavonoids was collected from both domestic and international sources. Priority should be given to flavonoids that have been confirmed by existing studies to be directly related to the pathogenesis or intervention effect of neurological diseases, with high quality and no contradictory results, clear chemical structures and definite metabolic pathways *in vivo*. Flavonoids that have no clear neurological association, have only been reported once and lack repeated verification or have a low level of evidence, and whose structures have not been resolved or whose metabolic processes are ambiguous should be excluded. After excluding studies with duplicate components and mechanisms, as well as outdated ones, a total of 190 core studies were finally screened and included from more than 15,000 literatures. These were systematically organized, categorized, and analysed across five dimensions: types of flavonoids, disease categories, experimental models, regulatory effects, and key targets.

**Results:**

Flavonoids can exert neuroprotective effects in the prevention and treatment of neurological diseases through multiple pathways, such as anti-inflammation, anti-oxidation, anti-apoptosis, and neurotransmitter regulation.

**Conclusion:**

Flavonoids exhibit clear pharmacological effects and favorable safety profiles in the prevention and treatment of neurological diseases, laying a foundation for the research and development of new drugs.

## 1 Introduction

The nervous system occupies a core position in the human body, dominating human regulation, information processing, maintaining balanced and coordinated movements, and advanced neural activities. However, this sophisticated system is vulnerable to genetic, infectious, traumatic, and other factors, leading to various neurological diseases. There are diverse kinds of disorders in the realm of nervous system, such as those connected with the brain, spinal cord, peripheral nervous system, neuromuscular junction, and muscles. These diseases are extremely common; statistics show that at least one-third of people will be affected by such diseases at some point in their lives (Owolabi et al., 2023; Feigin et al., 2020). The burden of death and disability caused by neurological diseases is increasingly becoming a major challenge in the global public health field, and there are signs that this burden will further increase in the next few decades (Feigin et al., 2020). For Alzheimer’s disease (AD), with the intensification of global population aging, the number of people diagnosed with this disease is expected to increase significantly in the next few decades, posing severe challenges to social and economic policies and elderly care (Creegan et al., 2015). At present, conventional treatment methods for neurological diseases mainly include drug therapy (Winston et al., 2024), surgical treatment (Ho et al., 2025), and rehabilitation therapy (Kang et al., 2025). Drug therapy can regulate neurotransmitters and relieve symptoms but has problems such as inability to cure the disease and obvious side effects (Moretti, 2025); surgical treatment, although suitable for specific conditions, faces the dilemmas of high risk, high cost, and difficulty in repairing overall nerve function (Ho et al., 2025); rehabilitation therapy helps functional recovery, but is restricted by individual patient conditions, with a long cycle, high cost, and difficulty in achieving complete regeneration and functional reconstruction of nerve tissue.

**Figure 1:**
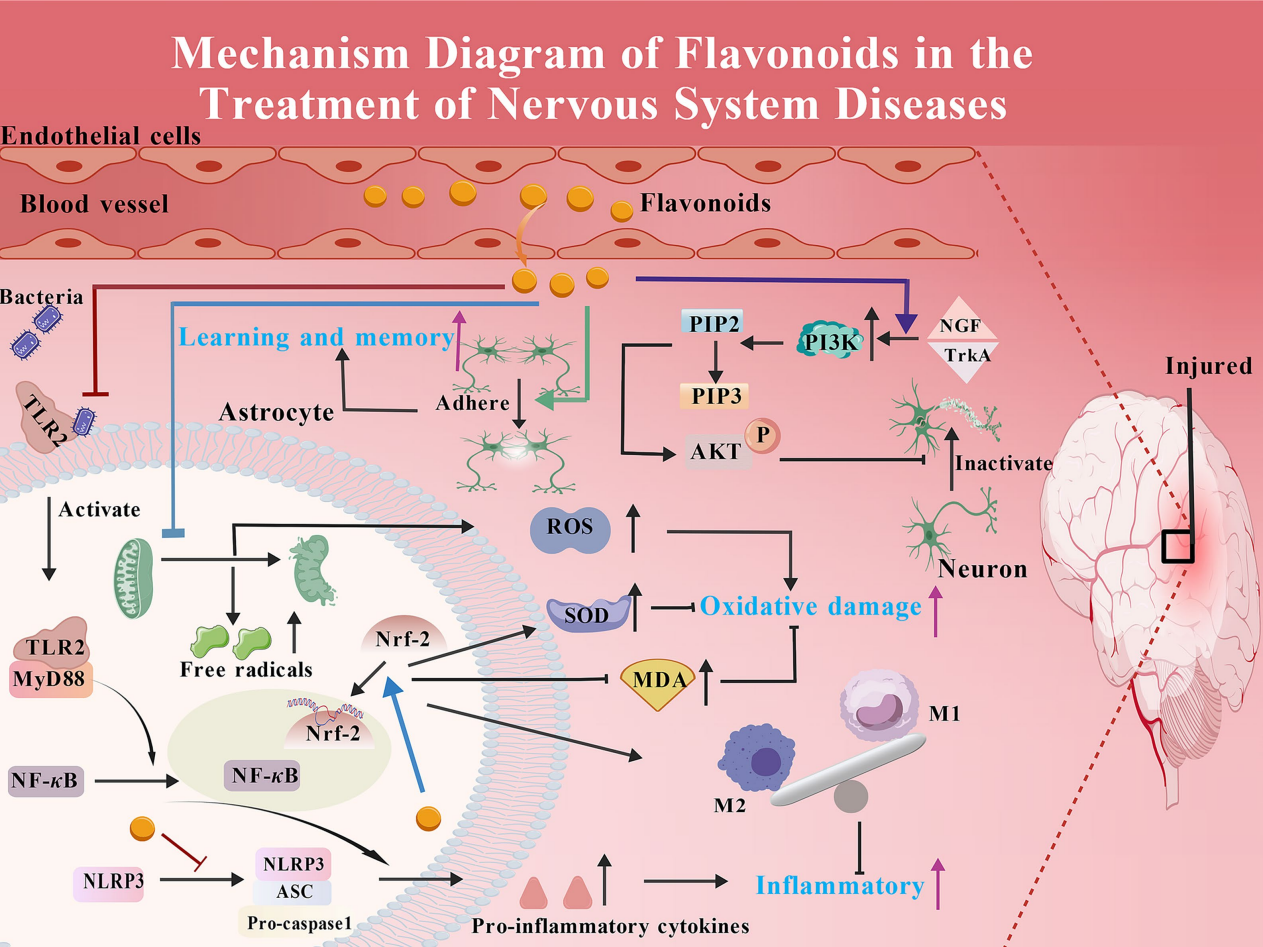
Schematic diagram of the multiple mechanisms by which flavonoids treat neurological disorders. Within the injured brain, flavonoids exert neuroprotective effects through multiple pathways, including anti-inflammatory and antioxidant actions, regulation of mitochondrial function, enhancement of synaptic plasticity, and inhibition of neuronal apoptosis.

The therapeutic effect of traditional Chinese medicine in treating neurological diseases has long been verified by thousands of years of clinical practice, and its historical basis can be traced back to systematic records in ancient medical classics. Zhang Zhongjing’s “Xiaochaihu Decoction” created in *On Typhoid and Miscellaneous Diseases* is a commonly used clinical prescription, which can effectively relieve neurological symptoms such as headache and irritability caused by exogenous febrile diseases and plays a significant role in regulating neurological dysfunction. Furthermore, in long-term clinical practice, traditional Chinese medicine has formed a complete treatment system for different neurological diseases. For example, for epilepsy, ancient physicians used “Dingxian Pills” to calm wind and resolve phlegm; for insomnia and amnesia, “Tianwang Buxin Dan” works by nourishing yin and blood, calming the mind, and improving intelligence. With the deepening of modern pharmacological research, it has gradually been found that the key strengths of traditional Chinese medicine in treating neurological diseases are often closely related to their active ingredients, among which flavonoids, as a class of natural ingredients widely present in medicinal plants, have become an important bridge connecting traditional Chinese medicine and modern medicine. Flavonoids are polyphenolic secondary metabolites present in plants, characterized by rich sources, diverse structures, and extensive biological activities (Yang et al., 2025). The pathogenesis of neurological diseases is complex, often involving multiple links such as nerve cell damage, inflammatory response, oxidative stress, and neurotransmitter imbalance. Flavonoids possess anti-oxidant and anti-inflammatory properties (Amin et al., 2025), and can achieve long-term protection of nerve cells by promoting the expression of nerve growth factors, inhibiting nerve cell apoptosis, and improving cerebral microcirculation. Compared with some chemical drugs, flavonoids have mild and long-lasting effects (Kukolj et al., 2025). Some flavonoids can boost the permeability of the blood–brain barrier (BBB) for better therapeutic outcomes (Al Hasan et al., 2025). Additionally, flavonoids also show unique advantages in improving nerve function (Rębas, 2025) and delaying disease progression (Bianchini et al., 2025; Liu et al., 2025), and are anticipated to have a more significant clinical role in neurodegenerative diseases, cerebrovascular diseases, and neuralgia (as shown in Figure 1).

The purpose of this review is to systematically organize the research development of flavonoids in preventing and treating neurological diseases, to further comprehend their action mechanisms and application advancements in various neurological ailments, and to define their benefits in disease prevention and treatment (Supplementary Table S1). Simultaneously, it objectively assesses the constraints of current research and anticipates future research and development directions in light of existing research findings, thus offering a comprehensive theoretical foundation and practical guidance for promoting in-depth research and clinical application of flavonoids in the area of neurological disease prevention and treatment.

## 2 The role of flavonoids in preventing and treating neurological diseases

### 2.1 Cerebral diseases

#### 2.1.1 Cerebrovascular diseases

Cerebrovascular diseases are a group of diseases that occur in the blood vessels of the brain, causing damage to brain tissue due to intracranial circulatory disorders. They mainly include ischemic cerebrovascular diseases and hemorrhagic cerebrovascular diseases. The etiology of ischemic cerebrovascular diseases is diverse, and the pathological mechanism is complex, but different etiologies may involve three basic pathological processes: vascular wall lesions, changes in blood components, and hemodynamic changes. Breviscapine, nobiletin, baicalein, etc., belong to flavonoids. Through the establishment of a rat model of middle cerebral artery occlusion (MCAO), it has been discovered that breviscapine can reduce cell apoptosis by inhibiting the expression of poly ADP-ribose polymerase-1 (PARP1) (Li et al., 2020a). By constructing a rat model of transient middle cerebral artery occlusion (t-MCAO), nobiletin offers a neuroprotective action on transient cerebral ischemia through anti-inflammatory and anti-apoptotic mechanisms (Yasuda et al., 2014). Baicalein reduces the release of pro-inflammatory cytokines and inhibits inflammatory responses by down-regulating the expression of NF-*κ*B, cyclooxygenase-2 (COX-2), prostaglandin E2 (PGE2), and lectin-like oxidized low-density lipoprotein receptor-1 (LOX-1). It can not only reduce oxidative stress by decreasing the content of MDA and increasing the activities of SOD, GSH, glutathione peroxidase (GSH-Px), and CAT but also reduce the level of AMPK, enhance the nuclear translocation of nuclear respiratory factor 2 (Nrf2), and enhance the endogenous antioxidant system (Yuan et al., 2020). The mechanisms of orientin (Wang et al., 2017), eupafolin (Chen X. et al., 2020), luteoloside (Li Q. et al., 2019), and total flavonoids of *Dracocephalum moldavica* L. (TFD) (Wu et al., 2018) in reducing oxidative stress and inflammatory response in the therapy of cerebral infarction are similar to those of baicalein. By constructing a rat model of hypoxic–ischemic encephalopathy (HIE) in neonates, apigenin can stimulate the PI3K/AKT/Nrf2 signaling pathway, inhibit cell apoptosis, reduce inflammatory response and oxidative stress, thereby exerting a neuroprotective effect on neonatal HIE (Fu et al., 2021). Baicalin has similar effects (Zhou et al., 2017). Quercetin, kaempferol, etc., belong to flavonols. Quercetin reduces the acetylation level of high-mobility group box 1 protein (HMGB1) by activating silent information regulator 1 (SIRT1), restricts the nucleocytoplasmic shuttling and secretion of HMGB1, and then inhibits the stimulation of the downstream TLR4/myeloid differentiation primary response gene 88 (MyD88)/NF-*κ*B signaling pathway, reduces the release of pro-inflammatory factors, alleviates neuroinflammatory response, reduces neuronal apoptosis, and facilitates the restoration of nerve function (Chen Z. et al., 2025). Kaempferol enhances the brain-derived neurotrophic factor (BDNF)-tyrosine kinase receptor B (TrkB)-PI3K/AKT signaling cascade, suppressing cellular apoptosis. This leads to a reduction in TUNEL-positive cells, attenuation of pro-apoptotic proteins, and upregulation of anti-apoptotic proteins. Additionally, it upregulates zonula occludens-1 (ZO-1) and occludin expression, reinforcing the BBB and facilitating the therapeutic effects against cerebral infarction (Zhang S. S. et al., 2022). Naringin belongs to dihydroflavonoids. It can activate the PI3K/AKT signaling pathway, promote the expression of p-AKT, increase the p-AKT/AKT ratio, up-regulate the expression of the anti-apoptotic protein B-cell lymphoma-2, down-regulate the expression of the pro-apoptotic protein Bax protein, inhibit nerve cell apoptosis, reduce cerebral infarction volume and brain water content, thereby treating cerebral infarction (Yang et al., 2020). The inflammatory mechanisms of hesperetin (Zhang J. et al., 2021) and taxifolin (Wang et al., 2006) in the treatment of cerebral infarction are similar to those of baicalein. Astilbin belongs to dihydroflavonols. It can inhibit the activation of the ROS-NLRP3 inflammasome axis, reduce ROS production, and inhibit inflammatory response, thereby treating cerebral infarction (Li et al., 2020b).

Hemorrhagic cerebrovascular diseases mainly include intracerebral hemorrhage and subarachnoid hemorrhage. A serious cerebrovascular condition known as intracerebral hemorrhage (ICH) is brought about by non-traumatic rupture of blood vessels within the brain parenchyma. This results in the accumulation of blood in the brain parenchyma. It is marked by an acute start, a high death rate, and a high rate of disability. Wogonin, baicalein, and breviscapine belong to flavonoids. By inducing ICH in mice through autologous blood injection, it was found that wogonin can enhance hematoma clearance after ICH and improve neurological function recovery by activating the PPAR-*γ* pathway and up-regulating the expression of anexelekto (Axl), macrophage erythroblast attached tyrosine kinase (MerTK), cluster of differentiation 36 (CD36), and lysosome-associated membrane protein 2 (LAMP2) (Zhuang et al., 2021). A collagenase-induced rat ICH model was established. The study found that baicalein could relieve brain damage following ICH by suppressing oxidative stress and NLRP3 inflammasome activation (Chen et al., 2022). The anti-inflammatory mechanisms of calycosin (Chen C. et al., 2020) and isoliquiritigenin (Zeng et al., 2017) in the treatment of intracerebral hemorrhage are similar to those of baicalein. Epigallocatechin gallate is a flavan-3-ol compound. Hao L. et al. (2024) in an *in vivo* experiment, induced a rat ICH model with collagenase IV, followed by intraperitoneal injection of epigallocatechin gallate. The results showed that epigallocatechin gallate significantly reduced cerebral edema, decreased iron content in brain tissue, and improved neurological deficits.

Subarachnoid hemorrhage (SAH) is an acute cerebrovascular disease, referring to the rupture of blood vessels at the base or surface of the brain, with blood flowing into the subarachnoid space. Luteolin, scutellarin, and baicalein belong to flavonoids. By injecting autologous arterial blood into the prechiasmatic cistern to simulate a rat SAH model, it was found that luteolin can significantly inhibit SAH-induced neuroinflammation, improve oxidative damage after SAH, restore the endogenous antioxidant system, and block NLRP3 inflammasome activation by activating the Nrf2 signaling pathway, thereby reducing neurological dysfunction and neuronal death (Zhang Z. H. et al., 2021). A rat SAH model was induced by endovascular perforation. The study found that scutellarin can significantly reduce cerebral vasospasm after SAH and can enhance the production of cerebrovascular endothelial nitric oxide synthase (eNOS) by up-regulating the exocellular signal-regulated kinase 5-kruppel-like factor 2-endothelial nitric oxide synthase (Erk5-KLF2-eNOS) signaling pathway, thereby exerting a vasodilatory effect (Li et al., 2016). Using a SAH model in rats, baicalein may protect against brain injury after SAH by reducing oxidative stress and glutamate neurotoxicity (Kuo et al., 2013). Phloretin belongs to dihydrochalcones. Through an *in vivo* SAH model, it was found that phloretin can significantly improve the prognosis of SAH mice, reduce inflammatory response, and exert anti-inflammatory effects by inhibiting the toll-like receptor 2 (TLR2)/MyD88/NF-*κ*B signaling pathway, thereby reducing neurological damage after SAH (Hao X. et al., 2024). Proanthocyanidin belongs to flavan-3-ols. By injecting autologous arterial blood into the cerebellomedullary cistern to induce a rat SAH model, proanthocyanidin showed antioxidant, anti-apoptotic, and anti-necrotic properties, helping to reduce oxidative damage and promote vasodilation (Yilmaz et al., 2018).

#### 2.1.2 Brain organic lesions

Intracranial infectious diseases mainly include encephalitis and meningitis. Encephalitis refers to inflammatory lesions caused by pathogens invading the brain parenchyma. The vast majority of cases are caused by viruses, but it can also be induced by infections with bacteria, fungi, spirochetes, rickettsiae, parasites, etc. Some cases may be allergic diseases. Luteolin, chrysin, baicalin, etc., belong to flavonoids. Luteolin can enhance the production of antiviral type I interferons and activate the cytoplasmic DNA-sensing cGAS-STING pathway. It can also directly bind to the active substrate binding site and promote the oligomerization of cGAS. Through the above pathways, luteolin can inhibit weight loss, neurodegeneration, and neuroinflammation related to murine herpes simplex virus encephalitis caused by HSV-1 infection (Wang Y. et al., 2023). Baicalin can also produce anti-inflammatory effects through the above pathways (Chang et al., 2007). Kaempferol belongs to flavonols. It can inhibit the transcription of the IE180 gene in the mouse brain, thereby reducing the transcription levels of early genes (EPO and TK) and inhibiting the expression of latency-associated transcripts. Meanwhile, it can reduce inflammatory responses (Li et al., 2021). Amentoflavone is a natural biflavonoid compound. It can inhibit the phosphorylation of cofilin, a key regulator of filamentous actin (F-actin) dynamics, thereby reducing cofilin-mediated F-actin reorganization and the intracellular transport of HSV-1, which decreases the early infection of HSV-1. Thus, it exerts a therapeutic effect on encephalitis caused by HSV-1 (Li F. et al., 2019). Anthocyanins enhance cellular antioxidant capacity by upregulating both GSH and glutathione oxidized levels while activating key endogenous defense systems, such as Nrf2 and HO-1, to counteract glutamate-mediated oxidative stress. This reduces excitotoxicity, oxidative damage, and neuroinflammation, thereby playing a certain role in curing encephalitis (Shah et al., 2016).

Meningitis refers to infectious inflammation of the subarachnoid space and meninges, which can be caused by viruses, bacteria, protozoa, or fungi. Its main manifestations include fever, headache, vomiting, and neck stiffness. Severe cases may lead to sequelae such as cranial nerve palsy, blindness, limb paralysis, epileptic seizures, and intellectual impairment. It is most common among children. Baicalin, acacetin, diosmetin, etc., belong to flavonoids. Baicalin can downregulate the genes (*yidC, rpiJ, liuE*) involved in biofilm formation in *Acinetobacter lwoffii* (*A. lwoffii*) and upregulate the genes (*otsA, betB, mprA, rcsC-3, otsB, ahpC*) involved in oxidative phosphorylation and TCSs (Ma et al., 2024). Acacetin can exert a therapeutic effect on meningitis by inhibiting the pore-forming activity and oligomerization of pneumolysin (PLY), a virulence factor of *Streptococcus pneumoniae* (*S. pneumoniae*) (Li S. et al., 2020). Diosmetin can inhibit the expression levels of AKT, PI3K, MyD88, and NF-*κ*B, and reduce the number of TUNEL-positive apoptotic cells, thereby alleviating neuroinflammation and neuronal apoptosis and treating meningitis (Zhang et al., 2019). Quercetin, kaempferol, etc., belong to flavonols. Quercetin can reduce the expression of inflammatory cytokines and BBB permeability marker genes (*Mmp9*, *Vegf*, *Ang-2*, and *Et-1*), increase the expression of angiogenesis genes (*Sema4D* and *PlexinB1*), reduce parasite-induced tight junction disruption, and inhibit the activation of the parasite-induced PI3K/AKT/extracellular signal-regulated kinase (ERK) signaling pathway, thus exerting a therapeutic effect on meningitis (Sun et al., 2024). As for kaempferol, it can bind to the catalytic active site of PLY to interfere with its pore-forming activity, thus restraining PLY-mediated cytotoxicity. Moreover, it has the ability to decrease the peptidase activity of sortase A (SrtA) through occupying the active site of SrtA. Since PLY and SrtA are key anti-infective targets, kaempferol can treat meningitis caused by *S. pneumoniae* (Xu et al., 2023).

Brain tumors form when abnormal cell division occurs in the brain or the surrounding areas. Such tumors may emerge either from the brain itself or from adjacent tissues, including nerves, the pituitary or pineal glands, and the protective layers surrounding the brain. Primary brain tumors (such as gliomas, meningiomas, and pituitary tumors) begin in the brain, whereas metastatic brain tumors develop when cancer from elsewhere in the body spreads to the brain. Baicalein and luteolin (3ʹ, 4ʹ, 5, 7-tetrahydroxy flavone) belong to flavonoids. Zhang X. et al. (2024) treated human glioma cell lines with baicalein and discovered that baicalein restrains programmed cell death of glioma cells by elevating the expression of epidermal growth factor receptor (EGFR) and knocking down casitas b-lineage lymphoma (CBL). It also slows down the degradation of EGFR protein through the overexpression of leucine-rich repeat-containing G protein-coupled receptor 4 (oe-LGR4). Meanwhile, baicalein downregulates the LGR4-EGFR pathway to inhibit the multiplication of glioma cells and trigger their programmed cell death, thereby treating glioma (Zhang X. et al., 2024). Hu et al. (2020) treated patient-derived glioma stem cells (GSCs) with luteolin and found that luteolin suppresses the exudation of transforming growth factor-beta 1 (TGF-*β*1) and the polarization of M2 macrophages in tumor-associated macrophages (TAM) (Zong et al., 2024). Quercetin is a flavonol compound. By activating the microRNA-197 (miR-197)/insulin-like growth factor binding protein 5 (IGFBP5) cascade, quercetin suppresses meningioma cell proliferation and enhances apoptosis. This mechanism involves reducing Bcl-2 levels and elevating Bax expression, ultimately exerting therapeutic effects against meningioma (Hu et al., 2020).

Traumatic brain injury (TBI) involves head trauma from physical impact that commonly causes substantial neurological impairment. Permanent functional limitations vary in severity according to the injury’s anatomical distribution (focal or diffuse patterns). Regional brain injuries can cause distinct symptoms: focal symptoms include abnormalities in locomotion, sensibility, speech, vision, hearing, etc., while diffuse brain damage often affects memory, sleep, or causes confusion and coma. Wogonin is a natural flavonoid compound. In a mouse model of intracerebral hemorrhage treated with wogonin, the expression of Axl, MerTK, CD36, and LAMP2 in perihematomal microglia and BV2 cells is upregulated through the PPAR-*γ* pathway. This promotes phagocytosis, accelerates hematoma clearance, and facilitates the recovery of neurological functions. Additionally, wogonin can reduce the expression of pro-inflammatory cytokines [tumor necrosis factor-*α* (TNF-*α*), interleukin (IL)-1*β*] and pro-oxidative enzymes after intracerebral hemorrhage, significantly alleviating inflammatory and oxidative stress responses in the mouse model of intracerebral hemorrhage (Zhuang et al., 2021).

Demyelination refers to damage to the myelin sheath after its formation. Demyelinating diseases mainly include multiple sclerosis and neuromyelitis optica. Apigenin, a flavonoid compound, can exert significant anti-neuroinflammatory effects by inhibiting inflammatory pathways like mitogen-activated protein kinase (MAPK), regulating the phenotype of immune cells, increasing regulatory T cells (Treg) (Charrière et al., 2024). Icariin is a flavonol compound. Experiments demonstrated that it markedly lowered the levels of NO, H₂O₂, MDA, while increasing the levels of SOD, CAT, glutathione peroxidase (GPx), IL-10. Icariin shows potential as a therapeutic agent for demyelination disorders (Song et al., 2024). Calycosin, a type of isoflavone, can upregulate the expression of Nrf2 in H₂O₂-induced mouse brain astrocyte C8D1A cells and in astrocytes of demyelination model mice induced by cuprizone (CPZ). Additionally, calycosin treatment can lessen oxidative stress and enhance the expression of antioxidants in CPZ-treated mice and astrocytes under H₂O₂-induced oxidative stress, suggesting its potential therapeutic role in multiple sclerosis (Chen et al., 2024).

#### 2.1.3 Neurodegenerative diseases

Neurodegenerative disorders represent a diverse category of progressive, age-related chronic illnesses (Jayawickreme et al., 2024). These conditions are marked by the progressive degeneration of neuronal architecture and functionality, eventually causing neuronal death, central nervous system dysfunction, and subsequent deterioration of cognitive, motor, and physiological processes (Izrael et al., 2025). AD, the most common etiology of dementia, is a chronic, progressive neurodegenerative condition with complex pathogenesis. Its clinical manifestations include progressive cognitive impairment and behavioral deficits (Xia et al., 2023), such as memory disorders, aphasia, apraxia, agnosia, and visual–spatial impairment. In addition, patients may experience a decline in abstract thinking and computing ability, accompanied by changes in personality and behavior. Nobiletin, isoorientin, chrysin, etc., belong to flavonoids. Nobiletin can improve short-term memory impairment and associative memory impairment in olfactory-bulbectomized (OBX) mice (Nakajima and Ohizumi, 2019). Chrysin (Singh Gautam et al., 2025) and isoorientin (Zhao Y. et al., 2024) can exert antioxidant effects by reducing MDA/ROS levels and increasing SOD/GPx levels. Chrysin can also downregulate the protein expression of IL-17 receptor A (IL-17RA), activator 1 (Act1), and tumor necrosis factor receptor-associated factor 6 (TRAF6), inhibit the phosphorylation of NF-*κ*B, and reduce inflammatory factor levels. Icariin, myricetin, hyperoside, etc., belong to flavonols. An AD model was established using APP/PS1 transgenic mice, which were then treated with icariin. Studies have shown that icariin can boost the expression of HMG-CoA reductase degradation 1 (HRD1), promote the total ubiquitination and K48-linked polyubiquitination of amyloid precursor protein (A*β*PP), and accelerate its degradation through the proteasome. Icariin can also reduce the co-localization of A*β*PP with the early endosome marker early endosome antigen 1 (EEA1), inhibit its entry into endosomes for cleavage by *β*/*γ* secretases to generate A*β*, thereby reducing A*β* production and improving cognitive impairment in AD mouse models (Chen X. et al., 2025). Researchers established an AD model by injecting A*β*1-42 and found that hyperoside can enhance the adenylate cyclase (AC)/protein kinase A (PKA) signaling pathway, improve A*β*1-42-induced memory impairment, and restore passive avoidance and object recognition abilities (Yi et al., 2022). Quercetin (Feng et al., 2024) and isorhamnetin (Wei et al., 2022) can inhibit the production of pro-inflammatory factors, exerting anti-inflammatory effects. Both quercetin and avicularin (Li Z. et al., 2024) can reduce the oxidative reactions induced by A*β*. Hesperidin, naringin, etc., belong to dihydroflavonoids. When mice were treated with hesperidin, it was found that hesperidin can reduce A*β* oligomers, alleviate A*β*-induced neurotoxicity, and reduce the overactivation of microglia (labeled by Iba-1) and astrocytes (labeled by GFAP) (Han et al., 2025). In the hippocampus, A*β* can induce the activation of COX-2 and Bax, and inhibit Bcl-2, cAMP response element binding protein (CREB), BDNF, and TrkB, while naringin can attenuate these effects of A*β* (Choi et al., 2023). Safflower yellow pigment belongs to chalcones. An AD model in mice was induced by scopolamine (SCOP), sodium nitrite (NaNO₂), and ethanol, followed by treatment with safflower yellow pigment. It was found that safflower yellow pigment can upregulate the expression of N-methyl-D-aspartate receptor 2B subunit (NMDAR 2B), phosphorylated calmodulin-dependent protein kinase II (p-CaMKII), phosphorylated cyclic adenosine monophosphate response element binding protein (p-CREB), BDNF, phosphorylated tyrosine receptor kinase B (p-TrkB), post synaptic density protein 95 (PSD95), and synaptic vesicle glycoprotein 2A (SV2A), activating the CREB/BDNF/TrkB pathway. Thus, it treats AD by resisting oxidative stress, promoting synaptic plasticity, and protecting neural morphology (Qi et al., 2025).

Parkinson’s disease (PD) is the second most prevalent neurodegenerative disorder after AD, clinically presenting with both motor and non-motor manifestations (Yasuda et al., 2014). One study suggests that the number of people with Parkinson’s disease globally will be 11.894 million in 2021, and is expected to grow to 25.2 million in 2050 (95% uncertainty range: 21.7 million to 30.1 million) (Su et al., 2025). Baicalin, apigenin, orientin, baicalein, gardenin A, etc., belong to flavonoids. In a mouse model of PD, it was found that baicalin can inhibit the phosphorylation of nuclear factor-kappa B p65 subunit (NF-*κ*Bp65), reduce the expression of NLRP3 and its related components, decrease the levels of caspase-1 and IL-1 (Huang et al., 2024). Apigenin exerts its effects by inhibiting neuroinflammation and oxidative stress (Anusha et al., 2017). Orientin alleviates neuroinflammatory responses, protects neurons from inflammatory damage, and relieves PD by inhibiting the relevant signaling pathway (Vasudevan Sajini et al., 2024). In a rat model of PD, researchers found that baicalein can reduce the expression of arachidonate 15-lipoxygenase, effectively alleviate motor disorders and tremor in rats (Kong et al., 2024). Diosmin (Habib et al., 2022) can exert anti-inflammatory effects and inhibit oxidative stress. Hack W and other researchers used transgenic mice expressing the mutant human A53T variant of *α*Syn (A53TSyn mice) and found that gardenin A significantly reduced the expression level of phosphorylated *α*Syn in mice, increased TH expression, and alleviated PD (Hack et al., 2024). Icaritin, morin, and isorhamnetin belong to flavonols. Wu et al. (2024) conducted a study where they injected rotenone (ROT) under the skin to induce a PD model in rats. Icaritin increased the number of synapse-rich cells (SRCs), improved neuroinflammation and facilitated the functional recovery of astrocytes (Wu et al., 2024). Wang Z. et al. (2023) used MPTP to induce a PD model in mice, using Neuro-2a and CF7 cells, and found that morin increased the expression of PTEN-induced kinase 1 (PINK1)/parkin RBR E3 ubiquitin protein ligase (Parkin), activated the AMPK/unc-51 like autophagy activating kinase 1 (ULK1) pathway, enhanced mitophagy without affecting mitochondrial membrane potential, promoted the nuclear translocation of transcription factor EB (TFEB), and induced autophagy. Qin et al. (2025) constructed a cellular model of PD by inducing SH-SY5Y cell damage with 6-hydroxydopamine. They found that isorhamnetin can target FOSL1 to activate the AKT/mammalian target of rapamycin (mTOR) signaling pathway, thereby reducing cell damage and alleviating PD. Sanggenol L and others belong to dihydroflavonoids. Zhao N. et al. (2024) used human neuroblastoma SK-N-SH cells to construct a PD model induced by rotenone. Sanggenol L can inhibit the expression of Bax, cytochrome c (Cyt-c), and caspase-12, -9, -3 proteins, while upregulating the expression of Bcl-2, inhibiting cell apoptosis, improving cell viability, and alleviating PD.

Huntington’s disease (HD) is also a type of neurodegenerative disease. The typical clinical triad of HD includes: (1) progressive movement disorders; (2) progressive cognitive impairment eventually leading to dementia; (3) psychiatric disorders, including depression, anxiety, apathy, compulsive behaviors, outbursts, addiction, and occasionally psychosis. Weight loss is a common feature (Gao et al., 2009). In western populations, the prevalence of HD is 10.6 to 13.7 per 100,000 people (McColgan and Tabrizi, 2018). Luteolin (3′,4′,5,7-tetrahydroxyflavone) is a type of flavonoid compound. Luteolin and its derivatives can significantly reduce cellular caspase-3-like activity and ROS levels, increase the nuclear level of phosphorylated (serine 40)-Nrf2, and enhance the transcriptional level of Nrf2/antioxidant response element (ARE). Meanwhile, luteolin derivatives can also enhance SOD activity, improve the level of glutamate-cysteine ligase catalytic subunit (GCLc) expression, and elevate the mRNA level of GCLc in mutant striatal cells, thereby slowing down or even preventing the progression of HD (Jayawickreme et al., 2024).

#### 2.1.4 Secondary pathological state

**Figure 2:**
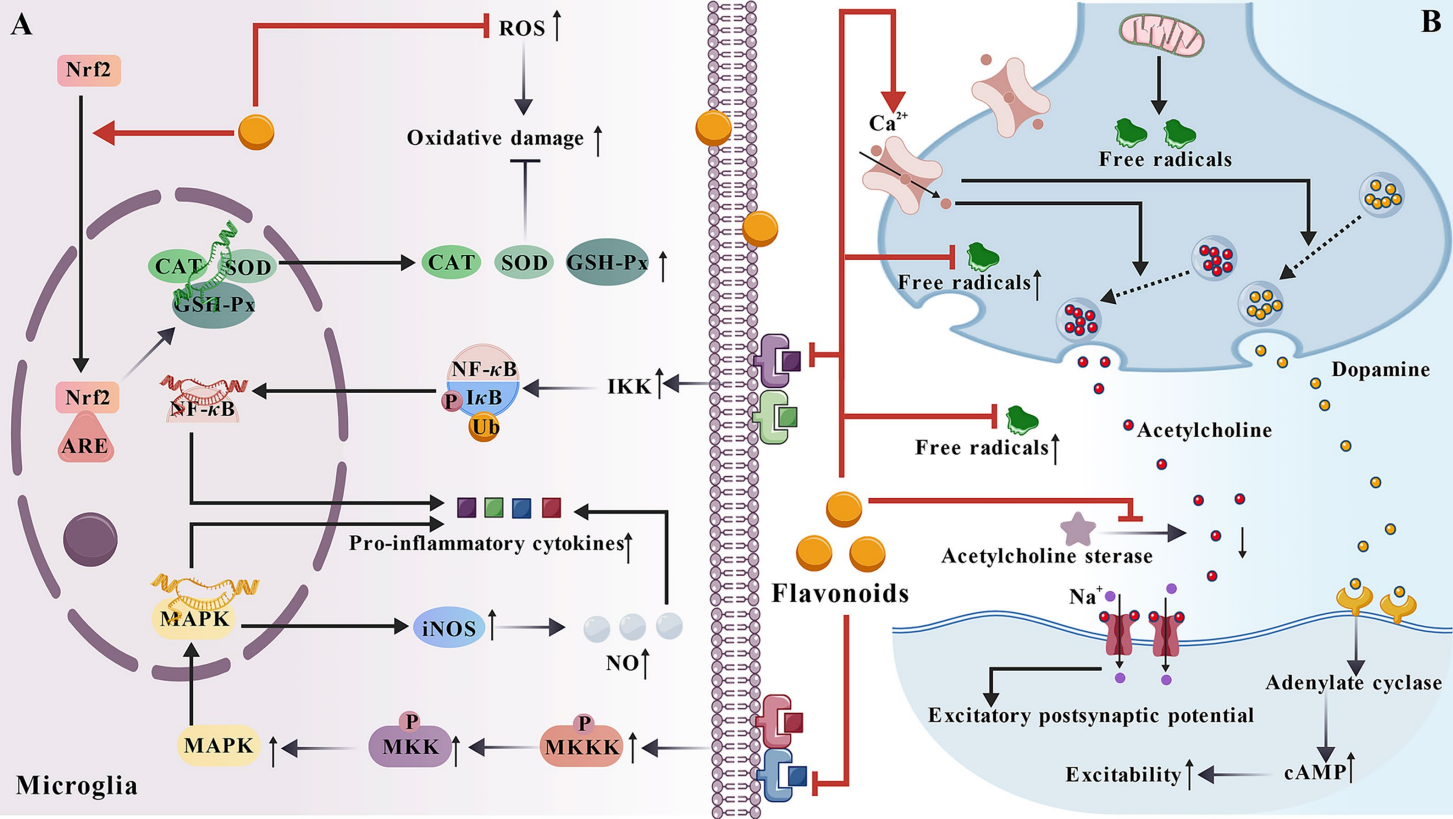
The mechanism by which flavonoids improve cerebral diseases. **(A)** In microglia, flavonoids can alleviate inflammatory responses. It can also eliminate ROS, upregulate the expression and activity of antioxidant enzymes, and reduce oxidative damage. **(B)** At the synapse, flavonoids can alleviate the damage to neurons caused by oxidative stress. Some flavonoids can promote calcium ion influx and the release of neurotransmitters. Flavonoids can also prevent the hydrolysis of acetylcholine in the synaptic cleft, enhance acetylcholinergic neural transmission, and make neural signal transmission more efficient.

Cerebral edema is a pathological state characterized by an increase in water content within the brain parenchyma, leading to brain tissue swelling. Hydrocephalus, on the other hand, is a pathological state caused by abnormal cerebrospinal fluid (CSF) circulation, which results in the accumulation of CSF in the ventricular system and subsequent ventricular dilatation. Luteoloside, apigenin, and chrysin belong to the flavonoid class. Researchers established a rat cerebral oedema model and found that luteoloside (Li Q. et al., 2019) and apigenin (Fu et al., 2021) suppress inflammatory responses by inhibiting the release and activity of inflammatory factors. They also found that chrysin (Rashno et al., 2023) and apigenin reduce oxidative stress by decreasing ROS production. This alleviates damage to the BBB and neural cells caused by oxidative stress, thereby alleviating brain oedema. Vitexin cut hypoxia-inducible factor-1*α* (HIF-1*α*) and vascular endothelial growth factor (VEGF), thereby treating BBB disruption and cerebral oedema (Min et al., 2015). Rutin, quercetin, and others belong to the flavonoid class. Using a rat brain oedema model, researchers found that rutin (Yuceli et al., 2020) reduces pyramidal cell degeneration and polymorphonuclear leukocyte (PMN) accumulation in brain tissue, thereby treating brain oedema caused by TBI in rats. Additionally, quercetin significantly reduced brain water content, effectively alleviating inflammation and brain oedema formation to counteract HACE (Patir et al., 2012). Naringenin is a dihydroflavonoid compound that inhibits the phosphorylated eukaryotic initiation factor 2*α* (eIF2*α*) in the endoplasmic reticulum (ER) stress pathway, activating transcription factor 4 (ATF4), and the activation of CCAAT/enhancer-binding protein homolog (CHOP) and apoptosis, thereby treating brain injury and reducing brain oedema (Deng et al., 2021). Isoliquiritigenin (4,2ʹ,4ʹ-trihydroxy chalcone) is a chalcone compound that can significantly reduce brain oedema by enhancing the activity of CAT and GSH-Px, thereby reducing brain infarct volume and neurological deficits (Zhan and Yang, 2006) (as shown in Figure 2).

### 2.2 Spinal cord diseases

**Figure 3:**
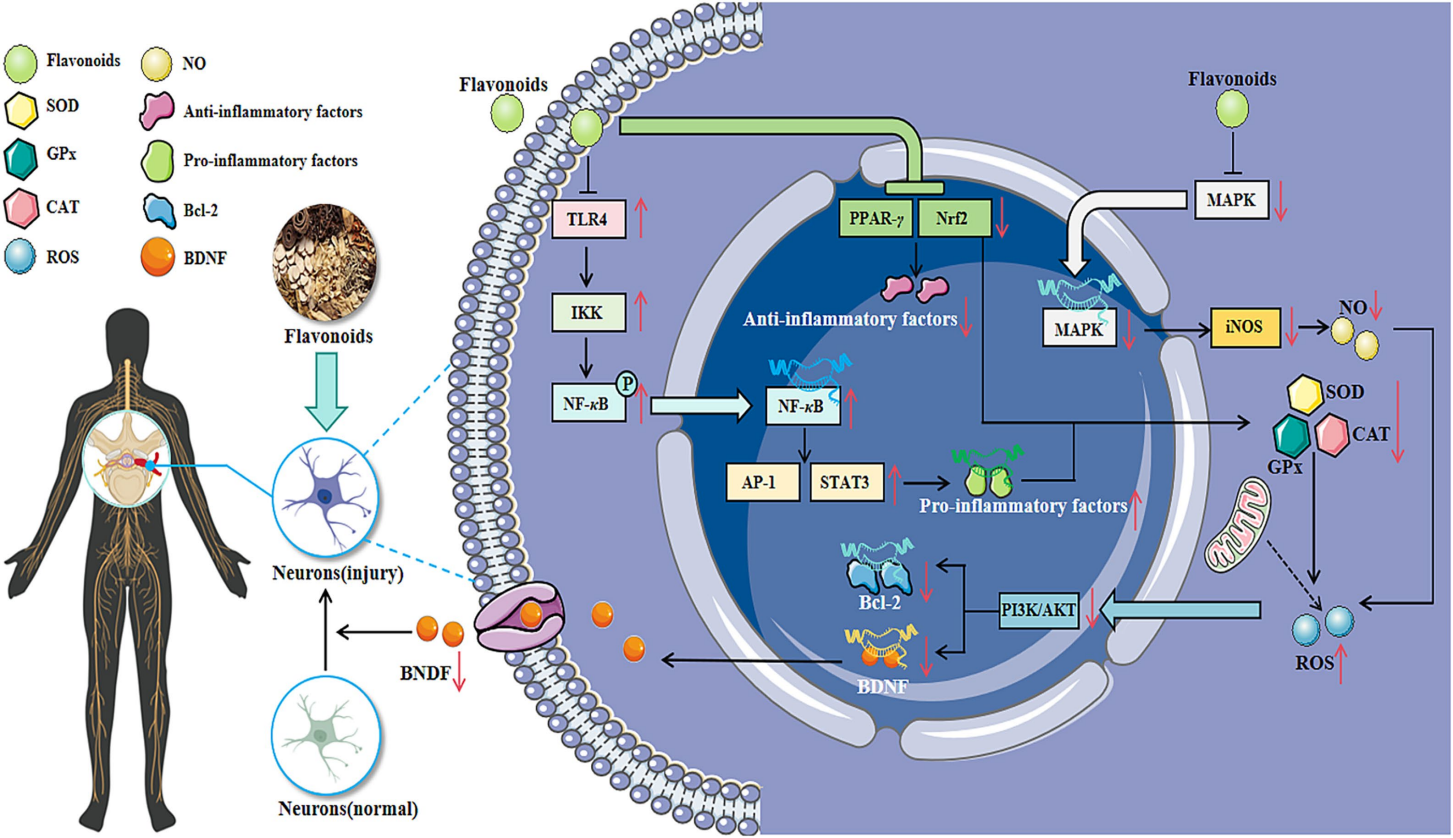
The related mechanisms of flavonoids in improving spinal nerve diseases. Flavonoids can exert multiple effects such as anti-inflammation, anti-oxidation, and regulation of neurotransmitters by regulating various signaling pathways. They are beneficial to the increase of neurotrophic factors, which are vital for the growth, development, survival and repair of neurons. Therefore, flavonoids have intervention potential for various nervous system diseases such as myelitis, spinal cord compression, and poliomyelitis.

Spinal cord diseases primarily include myelitis, spinal cord compression syndrome, and poliomyelitis, which can lead to impairments in motor, sensory, and autonomic nervous system functions (as shown in Figure 3).

“Myelitis” is an inflammatory lesion. Its clinical features include lower limb paralysis, sensory disturbances, and autonomic nerves system dysfunction. Myelitis can strike at any age, yet it exhibits a predilection for children and adolescents. Apigenin (Xie et al., 2023) belongs to the flavonoid class. Xie et al. (2023) used a paclitaxel-induced chemotherapy-induced peripheral neuropathy (CIPN) mice for their experiments. It was found that apigenin induces the nuclear translocation of Nrf2 and activates the Nrf2/ARE signalling pathway. Kaempferol, icariin, and isorhamnetin belong to the flavonol class. Kaempferol significantly alleviated oxidative stress and neuroinflammation after spinal cord injury by inhibiting NADPH oxidase 4 (NOX4) and NLRP3-mediated pyroptosis pathway (Liu et al., 2021). Icariin treatment significantly reduced the degree of phosphorylation of p38 mitogen-activated protein kinase (p38), cellular Jun (c-Jun), and mitogen-activated protein kinase kinase (MEK) in the brain tissue of mice with experimental autoimmune encephalomyelitis (EAE), thereby producing an anti-inflammatory effect (Cong et al., 2020). Isorhamnetin reduced the levels of MDA and 3-nitrotyrosine in the injured spinal cord, mitigated oxidative level (Chen et al., 2021). Tangeretin is a dihydroflavonoid compound. It can activate the Sesn2/Keap1 channel and provide a possible approach for the treatment of spinal cord injury (Peng et al., 2024). Puerarin is an isoflavone compound. Dai C et al. constructed a mice model of neuropathic pain. The study found that puerarin reduced inflammatory cell infiltration in the spinal cord, reduced the activation of inflammasomes in pro-inflammatory factors, and inhibited inflammatory responses in the spinal cord by enhancing Nrf2/glutathione peroxidase 4 (GPX4)-mediated antioxidant reactions, thereby alleviating neuropathic pain (Dai et al., 2024). Naringenin helps improve motor dysfunction and neuropathic pain after SCI by reducing neuroinflammation (Fakhri et al., 2022). Isoliquiritigenin is a chalcone compound and it can reduce the transcriptional expression of CCAAT enhancer binding protein beta (CEBPB), thereby alleviating spinal cord inflammation (Wang Z. et al., 2024).

Compressive myelopathy refers to a series of neurological dysfunction caused by compression of the spinal cord. Common causes include spinal stenosis, cervical spondylosis, spinal fractures, tumors, inflammation, and congenital spinal deformities. Among these, cervical spondylosis (such as cervical spondylosis) is the most common cause, primarily affecting individuals over 55 years of age, with a higher incidence in males than females. Compressive myelopathy can cause symptoms such as limb weakness, sensory abnormalities, and urinary and bowel dysfunction. In severe cases, paraplegia may occur. Imaging studies (such as MRI) are crucial for diagnosis, as they can reveal the location and extent of spinal cord compression. Treatment typically includes medication, physical therapy, or surgical decompression. The prognosis depends on the underlying cause and the timeliness of treatment. Quercetin is a flavonoid compound (Schültke et al., 2010). Hydroxysafflor Yellow A (HSYA) is a chalcone compound. Researchers established a rat spinal cord compression injury model and found that HSYA inhibits inflammatory responses by suppressing NF-*κ*B activation. Both HSYA and quercetin can alleviate oxidative stress by lowering MDA, myeloperoxidase (MPO), and NO levels while increasing SOD activity, suggesting their potential as therapeutic agents for spinal cord injury (Pei et al., 2017). epigallocatechin gallate, which is a natural catechin compound, also has certain functions. In a mouse femur model where MC57G fibrosarcoma cells were injected to induce bone cancer, epigallocatechin gallate lessened the neuroinflammation and pain behavior caused by bone cancer through suppressing TNF-*α* expression in the spinal cord (Li and Zhang, 2015).

Poliomyelitis (polio), an acute infectious disease mainly affecting the central nervous system (CNS), is caused by infection with poliovirus types I, II, and III. It is more common in children and is informally named “infantile paralysis” (Platt et al., 2014). The poliovirus enters the human body by binding to the immunoglobulin-like receptor cluster of differentiation 155 (CD155) and through endocytosis, infecting the CNS (Brandenburg et al., 2007). In some cases, patients may develop irregular distribution of limb muscle weakness and varying degrees of flaccid paralysis, leading to paralysis. Rarely, infection of bulbar neurons can cause respiratory paralysis or even respiratory arrest, threatening life. Currently, there is no specific antiviral drug for poliovirus. Treatment primarily involves symptomatic supportive care and promoting the recovery of neuromuscular function. Vaccination is the primary preventive measure. Polio has not yet been completely eradicated globally, and there remains a risk of cases caused by imported wild-type viruses. Hesperidin belongs to the dihydroflavonoid class. Bonies (1955) based on literature reviews and clinical experience, found that the combination of hesperidin and vitamin C can improve capillary integrity and function, reduce inflammation and oedema, and thus potentially alleviate polio symptoms. The article suggests the potential value of combining hesperidin and vitamin C in polio treatment, but lacks rigorous experimental data to support this claim. Nevertheless, the authors’ empirical observations provide a theoretical foundation for future research (Bonies, 1955).

### 2.3 Peripheral nervous system disorders

Peripheral nervous system disorders include cranial nerve disorders, spinal nerve disorders and peripheral nerve tumors.

Cranial nerve diseases refer to a group of neurological disorders characterized by dysfunction of the 12 pairs of cranial nerves. As a flavonoid compound, anthocyanidins can alleviate neuroinflammation by downregulating pro-inflammatory cytokine expression, suppressing microglial and astrocytic activation, enhancing autophagy, and limiting immune cell infiltration. They can also partially alleviate the chronicisation of trigeminal neuralgia, reduce neuroinflammation, inhibit glial cell activation, and promote autophagy, thereby delaying and alleviating trigeminal neuralgia. Additionally, they can promote the relief of motor symptoms and prevent their recurrence, offering a new potential strategy for treating trigeminal neuralgia (Magni et al., 2025).

Spinal nerve diseases refer to a series of neurological disorders caused by damage to the structure or function of the spinal cord. Baicalin belongs to flavonoids. Intraperitoneal injection of appropriate doses of baicalin into Diabetic neuropathic pain (DNP) rats revealed that baicalin could increase the mechanical withdrawal threshold of DNP rats, alleviate thermal hyperalgesia, prolong the thermal withdrawal latency, and prevent the development of Streptozotocin (STZ)-induced DNP. Meanwhile, baicalin could dose-dependently inhibit the upregulation of transient receptor potential vanilloid 1 (Trpv1) mRNA and protein expression in the dorsal root ganglion (DRG) of STZ-triggered DNP rats, thereby treating mononeuropathy (Li et al., 2018). Morin is a flavonol compound, which treats mononeuropathy through antioxidant and anti-apoptotic mechanisms (Jin et al., 2021). Icariin belongs to flavonols. Neuropathic pain rats were selected for the experiment. After the operation, the treatment group was given icariin by gavage daily. Icariin may exert therapeutic effects on mononeuropathy by inhibiting inflammation and cell apoptosis, providing a new potential drug option and theoretical basis for the treatment of neuropathic pain (Pokkula and Thakur, 2021). Narirutin and hesperidin belong to dihydroflavonoids. Adult male rats were used to establish a spared nerve injury (SNI) model, and 14 days after modeling, the rats were given narirutin by intrathecal injection. It was found that narirutin could specifically inhibit sodium channel subtype 1.7 (Nav1.7). This led to a reduction in Nav1.7 current, which in turn impeded the production and conduction of action potentials mediated by it, and decreased neuronal excitability; it inhibited veratridine-triggered nociceptor activity, reduced Ca^2+^ influx, and inhibited the transmission of nociceptive signals; it downregulated the expression of Nav1.7 in calcitonin gene-related peptide (CGRP)-labeled sensory neurons of the DRG, and attenuated the upregulation of Nav1.7, thereby reducing the generation and transmission of nociceptive signals, alleviating peripheral neuropathic pain, and further acting on mononeuropathy (Yang et al., 2022). A chronic constriction injury (CCI) model was developed via loosely tying the sciatic nerve of male rats. The results demonstrated that hesperidin can downregulate the co-expression of P2X purinoreceptor 3 (P2X3) receptor and neuronal nuclei (NeuN). Also, it could inhibit the phosphorylation of ERK1/2, so as to treat mononeuropathy (Tao et al., 2019). Genistein, an isoflavone compound, can alleviate lipopolysaccharide (LPS)-induced inflammatory damage in rat DRG neurons (DRGn) by exerting anti-inflammatory mechanisms (Zhou et al., 2022). Epigallocatechin gallate is a flavonoid compound. A mouse model of familial amyloidotic polyneuropathy (FAP) expressing human transthyretin (TTR) was established, and the mice were treated with epigallocatechin gallate by gavage. Epigallocatechin gallate significantly reduced the deposition of TTR in the gastrointestinal tract and peripheral nervous system, and also decreased the levels of FAP-related biomarkers such as endoplasmic reticulum stress markers and 3-nitrotyrosine. Epigallocatechin gallate has important roles and potential application prospects in the treatment of familial amyloidotic polyneuropathy (Ferreira et al., 2012).

Peripheral nerve tumors are a type of tumor. They originate in the peripheral nervous system (including nerve trunks, nerve plexuses, and nerve endings), typically classified into two main categories: benign and malignant. Benign peripheral nerve tumors primarily include neurofibromas and schwannomas, which generally grow slowly, have distinct borders, and offer favorable outcomes following surgical resection. Malignant peripheral nerve tumors include malignant peripheral nerve sheath tumors (MPNST) and neurofibrosarcomas, among others. These tumors grow rapidly, are highly invasive, prone to recurrence and metastasis, and are challenging to treat, often requiring a combination of surgery, radiation therapy, and chemotherapy. Hesperidin is a dihydroflavonoid compound. Semis et al. (2021) established a paclitaxel-induced peripheral neuropathy (PN) model in rats and administered hesperidin treatment, finding that hesperidin significantly reduced paclitaxel-induced oxidative stress markers, inhibited inflammatory factors, alleviated the upregulation of endoplasmic reticulum stress (ERS) markers, and exerted anti-apoptotic effects by regulating Bcl-2 family proteins and caspase-3 levels. Additionally, hesperidin improved PTX-induced thermal and cold hyperalgesia and reduced motor dysfunction (Semis et al., 2021).

### 2.4 Neuromuscular junctions and muscle diseases

**Figure 4:**
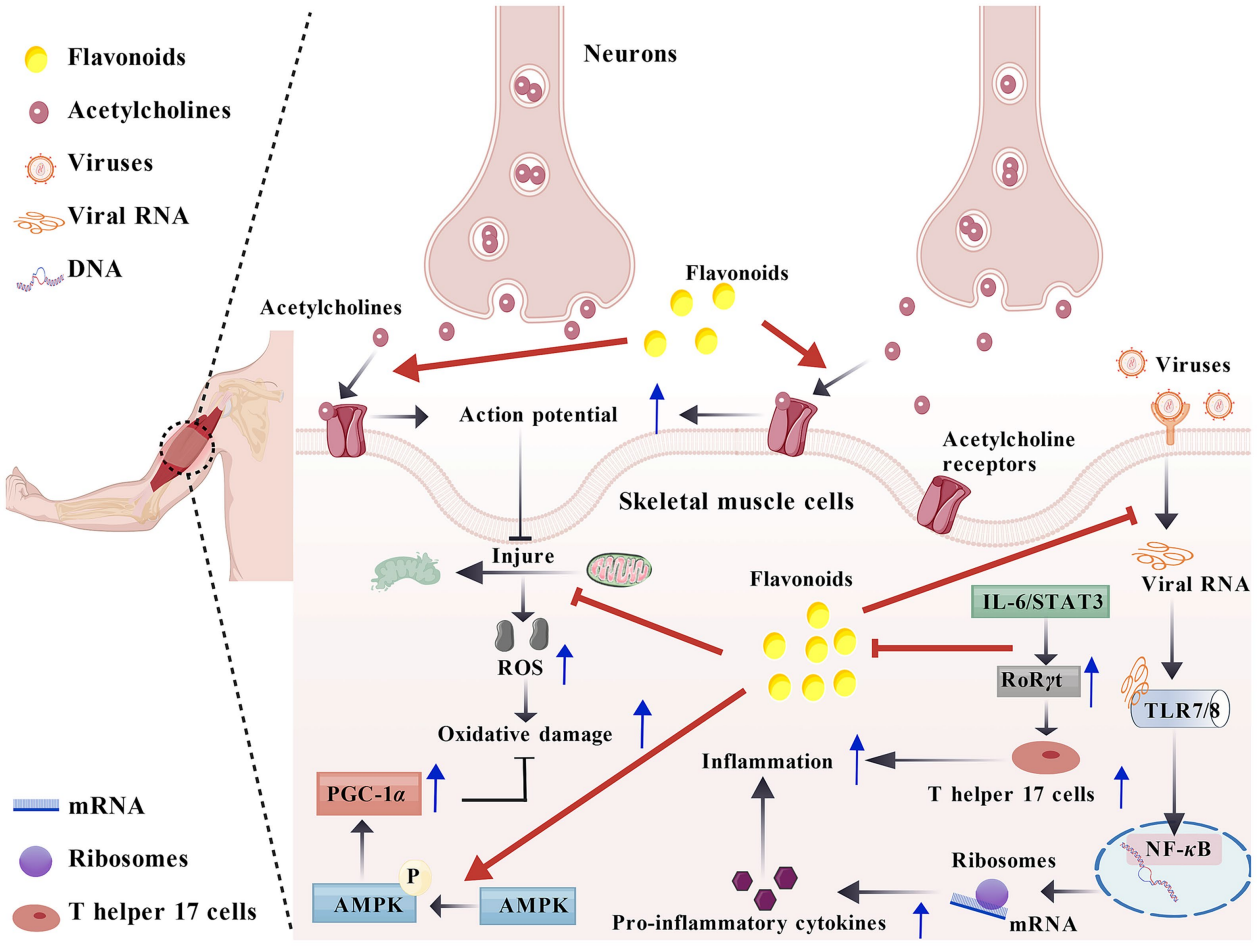
The therapeutic effects of flavonoids on neuromuscular diseases. Flavonoid compounds can not only accelerate the formation of action potentials, but also improve inflammatory responses by inhibiting the IL-6/STAT3 pathway and blocking the binding of viruses to receptors. They can also alleviate oxidative damage by inhibiting mitochondrial damage and promoting AMPK phosphorylation.

Neuromuscular junction and muscle diseases mainly include diseases such as myasthenia gravis and progressive muscular dystrophy (as shown in Figure 4).

Myasthenia gravis (MG) is an autoimmunological disease caused by dysfunction at the myoneural junction. The core cause of the disease is that the immune system mistakenly attacks the acetylcholine receptor (AChR) at the neuromuscular junction, preventing nerve signals from effectively transmitting to the muscles, thereby causing muscle weakness and fatigue. Symptoms worsen after physical activity and improve with rest. In critical presentations, it may affect the respiratory muscles, leading to breathing difficulties. In recent years, with advancements in diagnostic technologies, the global prevalence and incidence of MG have increased, but the mortality rate has gradually decreased. Khan H et al. explored the potential of flavonoids as acetylcholinesterase (AChE) inhibitors and their potential utility in MG therapy. The study used various plant-derived flavonoid compounds (such as quercetin, kaempferol, and chrysin) *in vitro* experiments. The inhibitory activity of flavonoids was assessed through AChE inhibition assays. It was found that flavonoids bind to the active site or peripheral anion site (PAS) of AChE, thereby preventing the hydrolysis of acetylcholine (ACh) and increasing the concentration of acetylcholine at the neuromuscular junction. Molecular docking studies revealed that certain flavonoids (e.g., quercetin) exhibit binding patterns with AChE similar to those of known AChE inhibitors (e.g., galantamine). AChE inhibitors enhance neuromuscular transmission by increasing the concentration of ACh in the neuromuscular junction, thereby improving the symptoms of myasthenia gravis. This study suggests that flavonoids, as AChE inhibitors, have the potential to improve neuromuscular transmission and may be of significant importance for the treatment of myasthenia gravis (Khan et al., 2018).

Progressive muscular dystrophy (PMD) represents a genetically diverse collection of inherited neuromuscular conditions unified by the hallmark features of gradually worsening muscle weakness and degeneration. Among these, duchenne muscular dystrophy (DMD) is the most prevalent type, arising from mutations in the DMD gene, with an X-linked recessive inheritance pattern. The occurrence rate is about 1 in 3,600 to 6,000 male infants, with a prevalence of approximately 1 in 3,853 in China. Globally, the prevalence of DMD varies significantly across different studies, ranging from 0.9 to 16.8 per 100,000 males and 0.7 to 7.7 per 100,000 in the general population. Overall, the pathogenesis of progressive muscular dystrophy is complex, with significant differences in clinical manifestations and severity among different types. Most types are rare diseases, requiring diagnosis through a combination of genetic testing and clinical presentation. Epicatechin is a natural flavonoid compound. Tapia-Curimil G et al. examined the impacts of epicatechin on inflammatory pathways and mitochondrial morphology in patients with PMD. The experimental model included one PMD patient and his healthy brother as a control. The patient consumed cocoa powder rich in epicatechin (100 mg/day) daily. The findings demonstrated that after epicatechin intake, the levels of inflammatory signaling proteins in the patient’s PBMCs partially returned to normal. Additionally, mitochondrial cristae length increased, and levels of proteins related to mitochondrial fusion/fission dynamics improved. These findings suggest that epicatechin may slow the progression of progressive muscular dystrophy by regulating inflammation and improving mitochondrial function (Tapia-Curimil et al., 2024). Cohen et al. (2023) investigated the effects of flavonoids (luteolin, apigenin, acacetin, luteolin 7-glucoside, apigenin 7-glucoside) on the effects of facioscapulohumeral muscular dystrophy (FSHD). FSHD is a common form of muscular dystrophy. The study found that these flavonoids can inhibit toxicity caused by DUX4 through an mTOR-independent mechanism, thereby protecting cells from DUX4-induced apoptosis. The experiment used the MB135-DUX4i myoblast model (MB135-DUX4 inducible myoblasts) to simulate the pathological process of FSHD by inducing DUX4 expression. A series of flavonoid compounds screened in the study were found to significantly inhibit DUX4-induced cellular toxicity at low micromolar concentrations, and this protective effect was associated with increased cellular autophagy activity. Additionally, these compounds did not negatively impact the expression of myogenic marker genes in patient-derived myotubes, suggesting their potential as therapeutic agents for FSHD (Cohen et al., 2023).

## 3 Challenges and prospects

**Figure 5:**
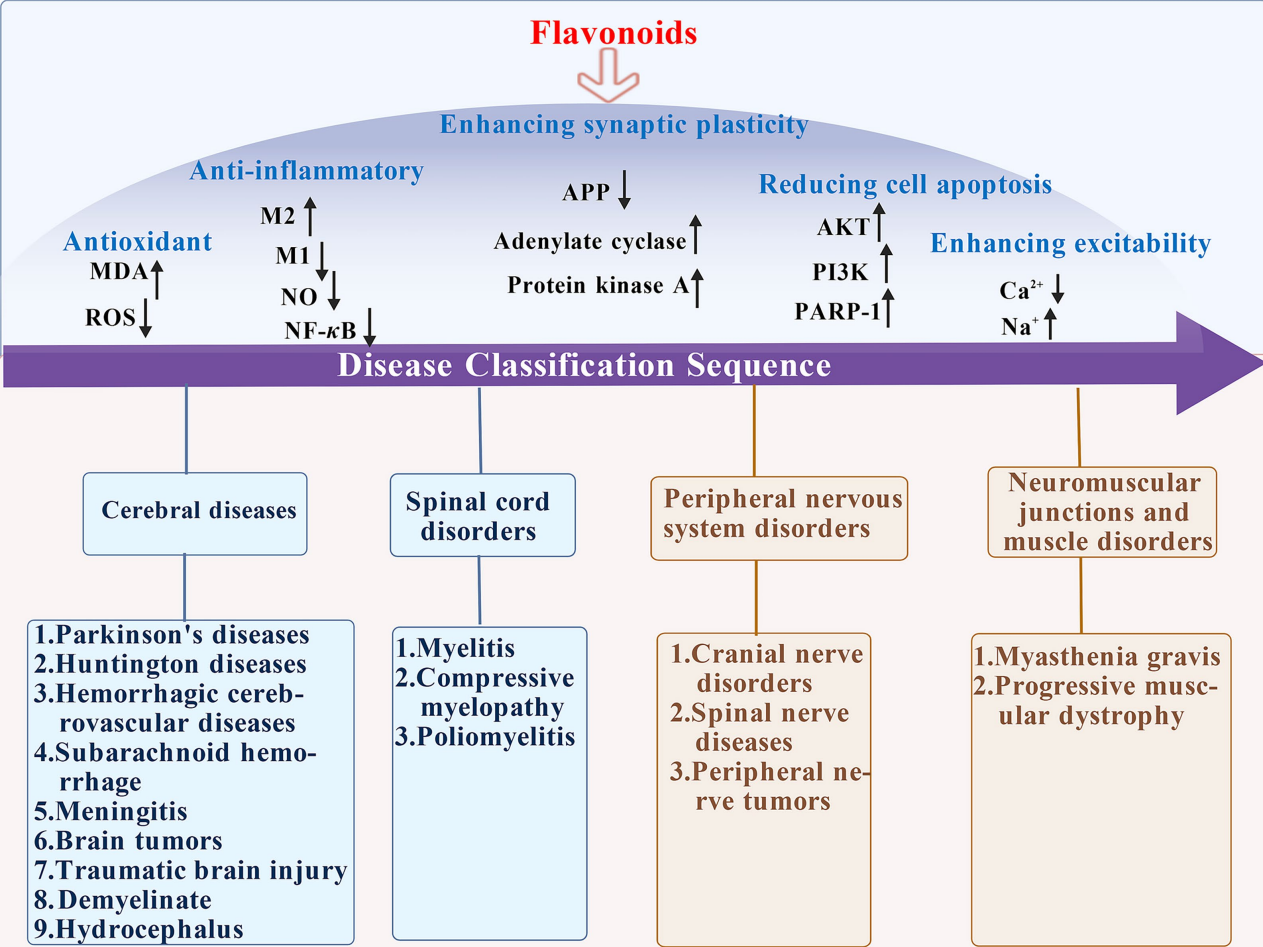
Flavonoids exert regulatory effects on inflammatory states, oxidative stress levels, and excitatory dysregulation associated with neurological disorders.

Flavonoids, as natural compounds widely present in medicinal plants, serve as a vital bridge linking traditional Chinese medicine with modern medicine. These compounds exert antioxidant effects by scavenging free radicals, inhibiting lipid peroxidation, and regulating the activity of antioxidant enzymes, thereby mitigating oxidative stress-induced damage to neural cells (Ding et al., 2015; Ye et al., 2025). Flavonoids can modulate the release of inflammatory mediators and the activity of inflammatory signalling pathways, thereby exerting anti-inflammatory effects and alleviating neuroinflammatory responses (Yang et al., 2024; Jin et al., 2019). It may also exert anti-apoptotic effects by suppressing the expression of apoptosis-related proteins and regulating the balance of apoptosis-regulating genes, thereby reducing neuronal cell death (Xu et al., 2025). Certain flavonoids may also promote the synthesis and secretion of neurotrophic factors (Xu et al., 2013), regulate neurotransmitter metabolism and signal transduction, thereby exerting neuroprotective and neuroregenerative effects (Yang et al., 2021; Faysal et al., 2025). These mechanisms collectively contribute to improving the pathological processes and symptoms of neurological disorders. The efficacy of flavonoids in treating neurological disorders is intrinsically linked to their unique chemical structure, embodying the molecular biological principle that “structure determines function.” The phenolic hydroxyl group enables it to scavenge hydroxyl radicals (Chen et al., 2002), and the number and position of these hydroxyl groups determine its antioxidant activity (Martínez Medina et al., 2017). Studies have demonstrated that the ortho-dihydroxyphenyl structure within the B ring of anthocyanins can inhibit COX-2 expression, conferring anti-inflammatory properties (Hou et al., 2005) (as shown in Figure 5). We have constructed a multi-dimensional matrix table (Supplementary Table S2), which aims to systematically integrate and present key information related to disease treatment research, thereby facilitating a more comprehensive evaluation and understanding of the potential value of different treatment strategies.

Nevertheless, flavonoids still require overcoming technical challenges in production preparation and ensuring resource quality. Chemical synthesis techniques (total/semi-total synthesis) utilize basic chemical raw materials to construct flavonoid aglycones and derivatives, thereby eliminating dependence on plant resources, streamlining processes, shortening cycles, and reducing costs (Bollikolla et al., 2022). Future efforts should integrate green synthesis with highly efficient catalytic systems, combining microbial synthesis (such as establishing microbial cell factories) with novel separation techniques like supercritical fluid extraction and high-speed counter-current chromatography to enhance the concentration of target compounds within extracts. Concurrently, through genomic analysis, standardisation of production and marketing, establishment of germplasm repositories, and enhanced molecular breeding, superior varieties shall be cultivated to safeguard resource quality (Fardoun et al., 2020). Additionally, market disorder can be addressed through fingerprint profiling and multi-indicator quantification (Winder et al., 2011), metabolomics and process monitoring (Xia et al., 2025), and industrialization technology integration for quality control and commercialization (Wang Y. et al., 2023). The water-insolubility of flavonoids may also limit their therapeutic efficacy in neurological disorders (Rębas, 2025). Addressing the water solubility issue of flavonoids may be approached through the following strategies. Firstly, hydrophilicity can be enhanced by modifying the molecular structure of flavonoids through salt formation modifications (Zhang Q. et al., 2022), glycosylation modifications (Kim et al., 2013), and the introduction of polar functional groups (Kongpichitchoke et al., 2015). Secondly, formulation design approaches such as nanomedicine technology, cyclodextrin inclusion complexation, and solid dispersion techniques also serve as potential solutions to this issue (Wang D. et al., 2022). Additionally, employing microorganisms (e.g., yeast, lactic acid bacteria) or enzymes (e.g., laccase) to bioconvert flavonoids into derivatives with enhanced water solubility offers another avenue (Feng and Cui, 2024). Finally, overcoming the bottleneck of poor water solubility in flavonoids can be achieved through process optimisation and improvements in extraction methods. Our research team has resolved the application bottleneck of baicalin’s poor water solubility by optimizing extraction processes to isolate a water-soluble baicalin magnesium salt (comprising two baicalin molecules and one magnesium ion) from traditional decoction of scutellaria baicalensis (Guan et al., 2023; Zhang L. et al., 2024). Compared to baicalin, baicalin magnesium exhibits superior water solubility and enhanced pharmacological activities, including anti-inflammatory, antioxidant, and hypolipidemic effects. Related research has secured invention patents in China, the United States of America, and the European Union (Liu et al., 2019).

To address the issue of low oral bioavailability of flavonoids in the treatment of neurological diseases, the following approaches can be adopted: first, structural modification can be used to optimize the physicochemical properties of flavonoids. Studies have shown that introducing substituents at specific positions on the flavonoid mother nucleus can significantly improve its metabolic stability and membrane permeability. For example, the acetylation modification of the 7-position hydroxyl group of quercetin can enhance its lipid solubility and increase the transmembrane transport rate in the Caco-2 cell model (Isika and Sadik, 2022). This structural modification reduces the binding sites with UDP-Glycosyltransferase (UGT) enzymes, thereby prolonging the retention time of the parent drug in the intestine (Noguchi et al., 2009). Second, nanocarriers (Tortella et al., 2023; Ruan et al., 2023; Wang C. et al., 2022) and prodrugs (Contin et al., 1996) can be employed to optimize absorption. Nanocarriers can avoid intestinal UGT metabolism. Examples include the construction of biomimetic brain-targeted liposomes, the utilization of the synergistic effect of self-emulsifying nanosystems, and the development of colon-targeted polysaccharide-based nanodrug delivery systems (Kumar et al., 2025). Prodrugs themselves have no or low biological activity, but are converted into active substances through *in vivo* metabolism. The purpose of this process is to increase the bioavailability of drugs, enhance targeting, and reduce drug toxicity and side effects. The prodrug R13 of 7,8-dihydroxyflavone, modified with an ester group, exhibits significantly improved oral bioavailability. In AD model mice, it effectively inhibits A*β* deposition and synaptic loss, while enhancing cognitive function (Chen et al., 2018). Additionally, the combined use of UGT inhibitors (such as atazanavir, curcumin, gemfibrozil, ketoconazole, and andrographolide) can increase the proportion of intact flavonoids in the intestine, thereby indirectly augmenting the source of the drug available in the brain (Ma et al., 2013). Beyond that, targeted intervention in the gut microbiota can regulate metabolic pathways. For instance, supplementation with Bifidobacterium R0175 increases the concentration of colonic SCFAs (e.g., butyric acid) (Li and Tang, 2025). SCFAs inhibit histone deacetylases (HDACs) and upregulate the expression of ZO-1, a tight junction protein of the BBB, which in turn improves the brain permeability of the drug (Wen et al., 2025). In a depressed mouse model, this intervention restored hippocampal serotonin levels to normal and reduced the immobility time in the forced swim test (Fung et al., 2017). Finally, the combined application of nanocarriers with enzyme inhibitors, as well as the integration of microbiota modulation with structural modification, also represent viable approaches to address this issue (Zhou et al., 2015). In the future, efforts to improve oral bioavailability may progress toward the development of multi-responsive intelligent delivery systems, the creation of novel excipients, and the advancement of combination therapy strategies (Liu et al., 2017).

Currently, several pharmaceutical preparations rich in flavonoids have been approved for marketing, indicated for the treatment of cognitive impairment and dementia-related conditions (Wang et al., 2016), cerebrovascular disorders (Chen, 2007), neurodegenerative diseases (Zhang et al., 2014), central nervous system infections (Sun and Wang, 2000), and spinal cord disorders (Feng and Zhang, 2000). Examples include Fufang Danshen Pian (Wang et al., 2016) for vascular cognitive impairment, Sanqi Tongshu capsule (Chen, 2007) for ischaemic cerebrovascular disease, and Bushen Tongluo capsule (Zhang et al., 2014) for advanced PD. Furthermore, multiple flavonoids are currently undergoing clinical trials, including quercetin (Gonzales et al., 2023) for treating AD (Identifier: NCT04063124), anthocyanins (Aarsland et al., 2023) for delaying cognitive decline in dementia high-risk populations (Identifier: NCT03419039), and genistein (Viña et al., 2022) for delaying the progression of prodromal AD (Identifier: NCT01982578), among others (Supplementary Table S3). However, flavonoids still face numerous challenges in the clinical translation process for neurological disorders. Core bottlenecks center on dose-related toxicity risks, BBB penetration barriers, incomplete toxicological mechanisms and evaluation systems, and prominent risks associated with drug interactions (Rębas, 2025). For instance, in animal-to-human dose conversion, the traditional body surface area method overlooks inter-species variations in metabolic enzyme activity, potentially leading to underdosing or toxicity in clinical applications (Löscher and Trenité, 2025). Furthermore, while certain flavonoids (e.g., quercetin, isoquercetin) demonstrate safety at low-to-moderate doses during short-term use, their long-term toxicity at high doses remains unclear. Safety assessments for special populations, including the elderly and those with hepatic or renal impairment, are also inadequate (Manjunath and Thimmulappa, 2022; Yao et al., 2021). To address dose-related limitations, pharmacokinetics and pharmacodynamics (PK/PD) models or physiologically based pharmacokinetic (PBPK) models can be constructed. These models enable cross-species dose translation from rats to humans and scale adult models to special subpopulations, providing a basis for drug doses in different populations and facilitating the development of precision medicine. For example, a study measured the concentration of relevant substances and prolactin levels in rats. It used the NONMEM software to build a PK-PD model, which was validated by visual predictive checks. *In vitro* data were incorporated to correct for species differences and predict human parameters. Subsequently, human data were used to simulate pharmacodynamic effects. Finally, clinical validation confirmed that the parameters were consistent with the predicted values, demonstrating the translational value of the PK-PD model (Chang et al., 2011). Another study constructed a PBPK model using data on *in vitro* metabolism, *in vivo* pharmacokinetics, and excretion in rats. By comparing the predicted and measured plasma concentration curves as well as key PK parameters, the study confirmed that the model could accurately describe the *in vivo* disposition process in rats. The model was then extrapolated to humans and its consistency was verified with clinical data from healthy volunteers. Finally, sensitivity analysis was conducted to identify core parameters, supporting extrapolation to special populations. This indirectly validated the stability of the “preclinical → human” correlation and demonstrated the translational capability of the PBPK model (Li et al., 2022). To address limitations in toxicity mechanisms and evaluation systems, cryo-electron microscopy, molecular modelling, and structural analysis techniques (Minicozzi et al., 2023) may be employed to investigate the action targets and toxicological mechanisms of flavonoid compounds, thereby refining toxicological assessment frameworks. Furthermore, metabolic regulation can also assist in mitigating toxicity risks. By inhibiting SULT1A1 via aminobenzoic acid (Rasool et al., 2019), the sulphate metabolism of flavonoids is reduced, thereby increasing the absorption of the parent drug. This approach enhances therapeutic efficacy while simultaneously diminishing toxicity arising from metabolic abnormalities.

The BBB, as the core physiological barrier regulating the transport of peripheral substances into the central nervous system, possesses selective permeability that directly determines whether flavonoid compounds can effectively reach central targets and exert biological effects. It thus constitutes the key functional barrier for flavonoids acting upon the central nervous system (Jiao et al., 2024). Flavonoid glycosides exhibit increased polarity due to their sugar moieties, leading to potent efflux by P-glycoprotein (P-gp) and BCRP in BBB endothelial cells, thereby limiting their clinical applicability. Enhancing BBB penetration efficiency may initially be achieved through receptor-mediated nanocarriers (Di Mauro et al., 2023; Barrejón et al., 2023; Cellot et al., 2022), thereby elevating drug concentrations within cerebrospinal fluid. A total of 24 studies on nanodrugs for the treatment of neurological disorders were retrieved from the clinicaltrials.gov database. Among them, 2 studies mentioned the opening of the BBB (Supplementary Table S4) and have completed the Phase 1 study stage. Another study has completed both the Phase 1 and Phase 2 study stages (Supplementary Table S4). Additionally, chemical modification strategies (e.g., methylation, etherification, conjugation of BBB-targeting groups) or targeted synthesis based on BBB penetration principles can optimise physicochemical properties (e.g., reducing water solubility, enhancing lipophilicity to match the BBB’s lipid bilayer characteristics) or evade recognition by BBB efflux transporters, thereby significantly improving target drug penetration efficiency (Narsinh et al., 2024). In addition, ultrasound can non-invasively open the BBB (Yang, 2025). In the clinicaltrials.gov database, with the keyword “ultrasound and BBB,” 61 research data can be retrieved. Among them, 3 are in the Phase 3 research stage (Supplementary Table S5). 4 have completed the Phase 1 and Phase 2 research stages (Supplementary Table S5). 3 have completed the Phase 1. Research phase (ClinicalTrials.gov ID, NCT03296852, NCT04804709, NCT04091503). Among the remaining items, 1 is in Phase 2, 5 are in Phase 1 and Phase 2, 7 are in Phase 1, and 10 are in the pre-research stage. Another 12 items are in an unknown state. Secondly, co-administration of piperazine can competitively bind P-gp/BCRP, reducing the potent efflux of P-gp and BCRP by BBB endothelial cells (Huang et al., 2018). Furthermore, acute neurological disorders may employ low-frequency focused ultrasound combined with microbubbles to transiently open the BBB, elevating intracerebral drug concentrations while restoring BBB integrity within 24 h (Hsu et al., 2022). Finally, central local administration bypassing systemic metabolism (e.g., intrathecal and intraventricular injections) (Li J. et al., 2024) and nasal mucosal delivery via the “nose-brain pathway” (Qiu et al., 2025) circumvent BBB penetration challenges.

Flavonoids may be combined with anti-Parkinson’s drugs (Singh et al., 2003), centrally acting analgesics (Hashemzaei et al., 2017), antidepressants (Dos Santos et al., 2025), cerebrovascular disease treatments (Stainer et al., 2019), and other specialised pharmaceuticals (Gonzales et al., 2023) to exert synergistic therapeutic effects. For instance, literature demonstrates that quercetin enhances the anticatatonic effect of levodopa combined with carbidopa in PD model rats by inhibiting catechol-O-methyltransferase (COMT) and MAO (monoamine oxidase) enzyme activity, thereby reducing dopamine degradation. This supports its potential as an adjunctive therapy for PD (Singh et al., 2003); luteolin (Hashemzaei et al., 2017) combined with low-dose morphine significantly enhances analgesic effects, demonstrating potential for “reducing toxicity while enhancing efficacy” in alleviating neuropathic pain; flavonoids such as quercetin (Stainer et al., 2019) and apigenin (Navarro-Núñez et al., 2008) synergistically enhance aspirin’s antiplatelet effects, strengthening its antithrombotic action. However, flavonoids also present certain challenges in drug combination therapy. For example, research indicates that certain flavonoid compounds inhibit CYP2D6 enzyme activity (Anderson et al., 2022; Wang M. et al., 2024). As CYP2D6 serves as a key metabolic enzyme for antidepressants (such as amitriptyline and fluoxetine), inhibition of its activity directly alters the metabolic rate and clearance efficiency of these antidepressants. However, existing research has not elucidated the precise binding site between flavonoids and the CYP2D6 enzyme, nor has it clarified the selective differences in this inhibitory effect across antidepressants of varying structural types. To address the aforementioned issues, comprehensive combination guidelines may be developed based on existing research data, clearly defining contraindications and safe dosage ranges for flavonoid compounds when used in combination with various medications. Subsequently, molecular biology techniques may be employed to elucidate the mechanisms by which flavonoids interact with key metabolic enzymes, analysing their selective differences in the metabolism of different types of antidepressants. Concurrently, pharmacogenomics should be employed to analyse metabolic variations in combination therapy across different genetic populations, thereby providing evidence for personalised treatment strategies and advancing the precision and safety of combination therapies (Mujinya et al., 2025). In addition, current research on flavonoids in neurological diseases mostly focuses on preclinical experimental studies conducted in cell and animal models. The differences between animal models and the clinical pathological state of the human body further limit the generalizability of research results to the human population. Existing clinical trial data also have issues such as small sample sizes and short follow-up periods, leading to translation barriers. In the future, it is necessary to strengthen translational medicine research, such as human trials, “model construction technologies” for human physiological/pathological microenvironments (Birtele et al., 2025), verification technologies for human cell lines (Ioghen et al., 2023) and primary cells (Whangviboonkij et al., 2023), multi-omics and AI prediction technologies (Wang and Zuo, 2025), as well as technologies combining fluorescent labeling and real-time imaging (Yan et al., 2017). These efforts aim to verify the efficacy and safety of flavonoids in the human population and supplement clinical data. By collating and comprehensively analyzing relevant literature, this article explores the impact of CYP2D6 genetic polymorphism on drug metabolism. To mitigate adverse interactions between flavonoids and CYP2D6 in polypharmacy for patients with neurological diseases, it proposes recommendations including genetic screening, therapeutic drug monitoring, selection of alternative drugs, patient education, close monitoring of adverse reactions, development of personalized treatment plans, and strengthening of multidisciplinary collaboration. Finally, we have summarized these *Challenges and prospects* into a simple and clear roadmap (Supplementary Figure S1).
