# Supplementary material for: The multi-pathway treatment of flavonoids as natural compounds in neurological diseases: achievements, limitations, and prospects
Source: Front Neurosci. 2025 Nov 5;19:1690170. doi: 10.3389/fnins.2025.1690170 (PMC12627070; doi:10.3389/fnins.2025.1690170)
Supplement: Supplementary file 1 [file Table_1.docx]

Supplementary Material

# Supplementary Table

**Table S1.** Pathway-semi-quantitative comparison in the treatment of neurological diseases

| **Pathway** | **Disease** | **Normal control group** | **Before treatment** | **After treatment** | **Reference** |
| --- | --- | --- | --- | --- | --- |
| PARP-1 pathway | Ischemic cerebrovascular disease | + | ++++ | ++ | [15] |
| NF-*κ*B, COX-2, PGE2, LOX-1 |  | + | ++++ | ++ | [17] |
| Oxidative stress, AMPK |  | + | ++++ | ++ |  |
| Nrf2 |  | ++ | + | +++ |  |
| PI3K/AKT/Nrf2 signaling pathway |  | ++ | + | ++ | [22] |
| TLR4 |  | + | ++++ | ++ | [24] |
| MyD88 |  |  |  |  |  |
| NF-*κ*B |  |  |  |  |  |
| PI3K/AKT signaling pathway |  | ++++ | +++ | +++ | [25] |
| BDNF-TrkB signaling pathway |  |  | + | +++ |  |
| PI3K/AKT signaling pathway |  | ++ | + | ++ | [26] |
| PPAR-*γ* pathway | Hemorrhagic cerebrovascular disease (ICH) | +++ | + | ++ | [30] |
| NLRP3 inflammasome activation |  | + | ++++ | ++ | [31] |
| Nrf2 signaling pathway | Hemorrhagic cerebrovascular disease (SAH) | + | ++ | +++++ | [35] |
| Erk5-KLF2-eNOS signaling pathway |  | ++ | + | +++ | [36] |
| TLR2/MyD88/NF-*κ*B signaling pathway |  | + | ++++ | ++ | [38] |
| AMPK-ULK1, PINK1-Parkin and TFEB pathway |  | ++ | + | +-+ | [39] |
| EGFR/LGR4 pathway | Brain tumors | ++++ | +++++ | ++ | [50] |
| TGF-*β*1/M2 macrophage polarization |  | - | ++++ | + | [51] |
| PPAR-*γ* pathway | Traumatic brain injury | +++ | + | ++ | [30] |
| Memory impairment pathway | Alzheimer's disease | +++ | + | ++ | [56] |
| Antioxidant and anti-inflammatory pathway |  | ++ | + | +++ | [57] |
| Amyloid precursor protein degradation pathway |  | ++ | + | ++ | [59] |
| Adenylate cyclase/protein kinase A pathway |  | ++ | + | ++ | [60] |
| Anti-neuroinflammation and anti-oxidative stress pathway | Parkinson's disease | ++ | + | ++ | [68] |
| Antioxidant response pathway | Huntington's disease | ++ | + | +++ | [53] |
| Anti-neuroinflammatory pathway via MAPK | Demyelinating diseases | ++ | + | ++ | [80] |
| Antioxidant pathway |  | ++ | + | ++ | [81] |
| Nrf2 antioxidant pathway |  | ++ | + | ++ | [82] |
| Anti-inflammatory pathway | Cerebral oedema | ++ | + | ++ | [20] |
| Anti-inflammatory and antioxidant pathway |  | ++++ | + | +++ | [22] |
| HIF-1*α* and VEGF inhibition pathway |  | ++ | + | ++ | [84] |
| Anti-inflammatory pathway |  | ++ | + | ++ | [85] |
| ER stress pathway inhibition |  | ++ | + | ++ | [87] |
| Antioxidant pathway |  | ++ | + | ++ | [88] |
| Nrf2/ARE signaling pathway | Myelitis | ++ | +++ | ++ | [89] |
| NOX4/NLRP3 pyroptosis pathway inhibition |  | ++ | +++ | ++ | [90] |
| Oxidative stress reduction pathway |  | ++ | + | ++ | [92] |
| Sesn2/Keap1 pathway activation |  | ++ | +++ | ++ | [93] |
| Nrf2/GPX4 antioxidant pathway |  | +++ | + | +++ | [94] |
| Anti-inflammatory pathway |  | ++ | + | ++ | [95] |
| CEBPB transcriptional regulation pathway |  | ++ | +++ | ++ | [96] |
| Inflammatory response (NF-*κ*B pathway) and oxidative stress pathway | Compressive myelopathy | ++ | +++ | ++ | [97] |
|  |  | ++ | +++ | ++ | [98] |
| Anti-inflammatory pathway through TNF-*α* inhibition |  | ++ | + | ++ | [99] |
| Anti-inflammatory and vascular integrity pathways | Poliomyelitis | ++ | + | ++ | [102] |
| Downregulating pro-inflammatory cytokine expression, suppressing microglial and astrocytic activation, enhancing autophagy, and limiting immune cell infiltration | Cranial nerve disorders | ++ | + | ++ | [103] |
| Inhibiting upregulation of Trpv1 mRNA and protein expression in dorsal root ganglion (DRG) | Spinal nerve diseases | ++ | +++ | ++ | [104] |
| Antioxidant and anti-apoptotic mechanisms |  | ++ | + | ++ | [105] |
| Inhibiting inflammation and cell apoptosis |  | ++ | + | ++ | [106] |
| Inhibiting Nav1.7 current and downregulating Nav1.7 expression in CGRP-labeled sensory neurons of DRG |  | ++ | + | ++ | [107] |
| Downregulating co-expression of P2X3 receptor and NeuN, inhibiting phosphorylation of ERK1/2 | Spinal nerve diseases | ++ | + | ++ | [108] |
| Anti-inflammatory mechanisms |  | ++ | + | ++ | [109] |
| Reducing TTR deposition and decreasing Familial Amyloidotic Polyneuropathy (FAP)-related biomarkers |  | ++ | + | ++ | [110] |
| Reducing oxidative stress markers, inhibiting inflammatory factors, alleviating ERS markers upregulation, exerting anti-apoptotic effects | Peripheral nerve tumors | ++ | + | ++ | [111] |
| Regulating inflammatory pathways and improving mitochondrial function | Progressive muscular dystrophy | ++ | + | ++ | [113] |
| Inhibiting DUX4-induced toxicity through mTOR-independent mechanism | Progressive muscular dystrophy | ++ | + | ++ | [114] |

**Note:** A "+" indicates the strength of the relevant pathway or the expression level of the protein. The greater the number of "+" symbols, the higher the strength of the pathway or the expression level of the protein.

**Table S2.** Supporting research on the neuroprotective effects of flavonoids and other substances through multiple targets and pathways.

| Disease | Compound | Pathway | Model/Species | Route/Dose/Regimen | Endpoints | Direction of Effect | Reference |
| --- | --- | --- | --- | --- | --- | --- | --- |
| Ischemic cerebrovascular disease | Breviscapine | PARP-1 pathway | Wistar rat middle cerebral artery occlusion (MCAO) model | *i.p.*, 20, 50, 100 mg/kg, 7 days | Cell apoptosis reduction | Reduce | [15] |
|  | Baicalein | NF-*κ*B, COX-2, PGE2, LOX-1 | Wistar rat MCAO model | *i.v.*, 2.5, 5, 10 mg/kg | Anti-inflammatory effects | Reduce | [17] |
|  |  | Oxidative stress, AMPK, Nrf2 |  |  | Antioxidant effects | Enhance |  |
|  | Apigenin | PI3K/AKT/Nrf2 signaling pathway | Neonatal hypoxic-ischemic encephalopathy model | *i.g.*, 20 mg/kg | Neuroprotection against apoptosis, inflammation, and oxidative stress | Enhance | [22] |
|  | Quercetin | TLR4/MyD88/NF-*κ*B signaling pathway | Neonatal ICR mice, MCAO model | *i.p.*, 120 mg/kg | Anti-inflammatory effects | Reduce | [24] |
|  | Kaempferol | BDNF-TrkB-PI3K/AKT signaling pathway | SD rat MCAO model | *i.p.*, 50 mg/kg | Anti-apoptotic effects | Enhance | [25] |
|  | Naringin | PI3K/AKT signaling pathway | SD rat MCAO model | *i.p.*, 6, 12, 25 *μ*g/mL | Anti-apoptotic effects | Enhance | [26] |
| Hemorrhagic cerebrovascular disease (ICH) | Wogonin | PPAR-*γ* pathway | Mouse intracerebral hemorrhage (ICH) model (autologous blood injection) | *i.p.*, 10 mg/kg | Hematoma clearance and neurological functional recovery | Enhance | [30] |
|  | Baicalein | NLRP3 inflammasome activation | Rat ICH model (collagenase-induced) | *i.p.*, 50 mg/kg, once every 12 hours for 3 days | Oxidative stress reduction | Reduce | [31] |
| Hemorrhagic cerebrovascular disease (SAH) | Luteolin | Nrf2 signaling pathway | Rat subarachnoid hemorrhage (SAH) model (autologous blood injection into prechiasmatic cistern) | *i.p.* 10, 30, 60, 90 mg/kg | Neuroinflammation inhibition, oxidative damage reduction | Reduce | [35] |
|  | Scutellarin | Erk5-KLF2-eNOS signaling pathway | Rat SAH model (endovascular perforation-induced) | *i.c.v.*, 100 mg/kg, 50 *μ*M | Cerebral vasospasm reduction | Reduce | [36] |
|  | Baicalein | Oxidative stress and glutamate neurotoxicity | Male Wistar rat SAH model | *i.p.*, 30 mg/kg, 6 days | Brain injury protection | Enhance | [37] |
|  | Phloretin | TLR2/MyD88/NF-*κ*B signaling pathway | Male C57BL/6J mouse SAH model | *i.g.*, 50 mg/kg, 3 days | Neurological damage reduction | Reduce | [38] |
|  | Proanthocyanidin | AMPK-ULK1, PINK1-Parkin and TFEB pathway | Rat SAH model (autologous arterial blood injection into cerebellar medullary cistern) | *i.g.*, 100 mg/kg, 5 days | Antioxidant, anti-apoptotic, anti-necrotic effects | Enhance | [39] |
| Brain tumors | Baicalein | EGFR/LGR4 pathway | Human glioma cell lines | *In vitro*, 80 *μ*M | Glioma cell proliferation, programmed cell death | Inhibit | [50] |
|  | Luteolin | TGF-*β*1/M2 macrophage polarization | Patient-derived glioma stem cells | *In vitro*, 60 *μ*M | TGF-*β*1 secretion, M2 macrophage polarization | Suppress | [51] |
| Traumatic brain injury | Wogonin | PPAR-*γ* pathway | Mouse intracerebral hemorrhage model | *i.p.*, 10 mg/kg | Phagocytosis, hematoma clearance, neurological function recovery | Enhance | [30] |
| Alzheimer's disease | Nobiletin | Memory impairment pathway | Olfactory-bulbectomized mice | *i.g.*, 50 mg/kg | Short-term memory and associative memory improvement | Improvement | [56] |
|  | Chrysin | Antioxidant and anti-inflammatory pathway | A*β*1−42-induced Alzheimer's disease (AD) model | *i.g.*, 25, 50, 100 mg/kg, 7 days | Antioxidant activity and reduced inflammatory factor levels | Improvement | [57] |
|  | Icariin | Amyloid precursor protein degradation pathway | APP/PS1 transgenic mice | *i.g.*, 60 mg/kg, 90 days | Reduced A*β* production and improved cognitive impairment | Improvement | [59] |
|  | Hyperoside | Adenylate cyclase/protein kinase A pathway | A*β*1-42-induced AD model | *s.t.i.*, 10, 30, 100 mg/kg, 3 days | Improved memory impairment and restored behavioral abilities | Improvement | [60] |
| Parkinson's disease | Baicalin | Neuroinflammation and oxidative stress pathway | Parkinson's disease (PD) mouse model | *i.g.*, 200 mg/kg | Inhibited NF-*κ*B p65 phosphorylation and reduced NLRP3 expression | Improvement | [68] |
|  | Morin | Mitophagy and autophagy pathway | MPTP-induced PD mouse model | *i.g.*, 12.5, 25 mg/kg | Increased PINK1/Parkin expression and promoted mitophagy | Improvement | [75] |
| Huntington's disease | Luteolin | Antioxidant response pathway | Mutant striatal cell model | *i.g.*, 25, 50, 75 100 *μ*M, 33 days | Reduced Caspase-3 activity and ROS levels, enhanced Nrf2 activity | Improvement | [53] |
| Demyelinating diseases | Icariin | Antioxidant pathway | Cuprizone-induced demyelination model in mice | *i.p.*, 50 mg/kg, 14 days | Reduced NO, H₂O₂, MDA levels; enhanced SOD, CAT, GPx, IL-10 | Improvement | [81] |
|  | Calycosin | Nrf2 antioxidant pathway | C8D1A cells and C57BL/6 mice model | *In vitro* and *i.g.*, 15, 30 *μ*M; 20 mg/kg | Upregulated Nrf2 expression and reduced oxidative stress | Improvement | [82] |
| Cerebral oedema | Luteoloside | Anti-inflammatory pathway | Rat cerebral oedema model | *i.p.*, 20, 40, 80 mg/kg | Suppressed inflammatory responses | Improvement | [20] |
|  | Apigenin | Anti-inflammatory and antioxidant pathway | Rat cerebral oedema model | *i.g.*, 20 mg/kg | Reduced oxidative stress and inflammation | Improvement | [22] |
|  | Vitexin | HIF-1*α* and VEGF inhibition pathway | Rat cerebral oedema model | *i.p.*, 30, 45, 60 mg/kg | Reduced BBB disruption and brain oedema | Improvement | [84] |
|  | Rutin | Anti-inflammatory pathway | Rat cerebral oedema model | *i.g.*, 50 mg/kg, 3 days | Reduced pyramidal cell degeneration and PMN accumulation | Improvement | [85] |
|  | Naringenin | ER stress pathway inhibition | Rat cerebral oedema model | *i.g.* 100 mg/kg, 3 days | Inhibited eIF2*α* phosphorylation and CHOP activation | Improvement | [87] |
|  | Isoliquiritigenin | Antioxidant pathway | Rat cerebral oedema model | *i.g.*, 5, 10, 20 mg/kg, 7 days | Enhanced CAT and GSH-Px activity; reduced brain infarct volume | Improvement | [88] |
| Myelitis | Apigenin | Nrf2/ARE signaling pathway | Chemotherapy-induced peripheral neuropathy mice | *i.g.*, 25, 50, 75 mg/kg, 16 days | Induced nuclear translocation of Nrf2 and activation of Nrf2/ARE pathway | Improvement | [89] |
|  | Kaempferol | NOX4/NLRP3 pyroptosis pathway inhibition | Spinal cord injury model | *i.g.*, 25, 50, 100 mg/kg, 10 days | Reduced oxidative stress and neuroinflammation | Improvement | [90] |
|  | Isorhamnetin | Oxidative stress reduction pathway | Spinal cord injury model | *i.g.*, 5 mg/kg | Reduced MDA and 3-nitrotyrosine levels | Improvement | [92] |
|  | Tangeretin | Sesn2/Keap1 pathway activation | Spinal cord injury model | *i.g.*, 20 mg/kg, 10 days | Activation of Sesn2/Keap1 channel | Improvement | [93] |
|  | Puerarin | Nrf2/GPX4 antioxidant pathway | Neuropathic pain mice | *i.p.*, 10 mg/kg, 7 days | Reduced inflammatory cell infiltration and inflammasome activation | Improvement | [94] |
|  | Naringenin | Anti-inflammatory pathway | Spinal cord injury model | *i.t.*, 5, 10, 15×10^3^ *μ*M, 20 *μ*L | Improved motor dysfunction and neuropathic pain | Improvement | [95] |
|  | Isoliquiritigenin | CEBPB transcriptional regulation pathway | Spinal cord injury model | *i.g.*, 20, 40 mg/kg, 4 days | Reduced CEBPB transcriptional expression | Improvement | [96] |
| Compressive myelopathy | Quercetin | Inflammatory response (NF-*κ*B pathway) and oxidative stress pathway | Rat spinal cord compression injury model | *i.p.*, 7.156 *mg*/kg | Alleviated oxidative stress by lowering MDA, MPO, NO levels; increased SOD activity | Improvement | [97] |
|  | Hydroxysafflor Yellow A |  |  | *i.p.*, 14 mg/kg, 7 days | Inhibited inflammatory responses; alleviated oxidative stress |  | [98] |
|  | Epigallocatechin gallate | Anti-inflammatory pathway through TNF-*α* inhibition | Mouse femur model with bone cancer | *i.p.*, 10, 25, 50, 100 mg/kg, 7 days | Reduced neuroinflammation and pain behavior | Improvement | [99] |
| Poliomyelitis | Hesperidin | Anti-inflammatory and vascular integrity pathways | Patient | *p.o.*, 600 mg/d | Improved capillary integrity and function; reduced inflammation and edema | Improvement | [102] |
| Cranial nerve disorders | Anthocyanidins | Downregulating pro-inflammatory cytokine expression, suppressing microglial and astrocytic activation, enhancing autophagy, and limiting immune cell infiltration | Experimental autoimmune encephalomyelitis model | *i.g.*, 53 mg/kg, 11 days | Alleviated neuroinflammation, inhibited glial cell activation, promoted autophagy, delayed and alleviated trigeminal neuralgia | Improvement | [103] |
| Spinal nerve diseases | Baicalin | Inhibiting upregulation of Trpv1 mRNA and protein expression in DRG | Diabetic neuropathic pain (DNP) rats | *i.p.*, 0.04 mg/kg, inject every 2 days for 49 days. | Increased mechanical withdrawal threshold, alleviated thermal hyperalgesia, prolonged thermal withdrawal latency, prevented development of STZ-induced DNP | Improvement | [104] |
|  | Morin | Antioxidant and anti-apoptotic mechanisms | Mononeuropathy model | *i.g.*, 50 mg/kg | Treated mononeuropathy | Improvement | [105] |
|  | Icariin | Inhibiting inflammation and cell apoptosis | Neuropathic pain rats | *i.g.*, 100 mg/kg, 21 days | Exerted therapeutic effects on mononeuropathy | Improvement | [106] |
|  | Narirutin | Inhibiting Nav1.7 current and downregulating Nav1.7 expression in CGRP-labeled sensory neurons of DRG | SNI model in adult male rats | *i.t.*, 20 *μ*g/20 *μ*L | Reduced neuronal excitability, inhibited nociceptive signal transmission, alleviated peripheral neuropathic pain | Improvement | [107] |
| Spinal nerve diseases | Hesperidin | Downregulating co-expression of P2X3 receptor and NeuN, inhibiting phosphorylation of ERK1/2 | The chronic constriction injury model in male rats | *i.p.*, 50 mg/kg, 14 days | Treated mononeuropathy | Improvement | [108] |
|  | Genistein | Anti-inflammatory mechanisms | Cells obtained from an LPS-induced inflammatory damage in rat DRGn | *In vitro*, 5, 10 *μ*M | Alleviated inflammatory damage | Improvement | [109] |
|  | Epigallocatechin gallate | Reducing TTR deposition and decreasing FAP-related biomarkers | FAP mouse model | *i.g.*, 100 mg/kg, 42 days | Significant reduction in TTR deposition, decreased levels of FAP-related biomarkers | Improvement | [110] |
| Peripheral nerve tumors | Hesperidin | Reducing oxidative stress markers, inhibiting inflammatory factors, alleviating ERS markers upregulation, exerting anti-apoptotic effects | Paclitaxel-induced peripheral nerve tumors model in rats | *i.g.*, 100, 200 mg/kg, 10 days | Improved PTX-induced thermal and cold hyperalgesia, reduced motor dysfunction | Improvement | [111] |
| Progressive muscular dystrophy | Epicatechin | Regulating inflammatory pathways and improving mitochondrial function | One progressive muscular dystrophy patient and his healthy brother | *p.o.*, 100 mg/d | Reduced inflammatory signaling proteins, increased mitochondrial cristae length and improved mitochondrial dynamics | Improvement | [113] |
|  | Luteolin,  Apigenin,  Acacetin,  Luteolin 7-glucoside,  Apigenin 7-glucoside, | Inhibiting DUX4-induced toxicity through mTOR-independent mechanism | MB135-DUX4i myoblast model | *In vitro*, 20 *μ*M | Protected cells from DUX4-induced apoptosis, increased cellular autophagy activity | Improvement | [114] |

### Table S3. Summary of clinical applications

| Clinical Stage/  Marketed | Ingredients | Effects | Number of Cases | Observation Duration | Observation Method | Observation Conclusions | Efficacy Evaluation | References | Identifier |
| --- | --- | --- | --- | --- | --- | --- | --- | --- | --- |
| Marketed | Ginkgo biloba leaf extract tablets,  flavone glycosides | Improving cerebral circulation and nerve cell metabolism, enhancing the learning process, and alleviating dementia symptoms as well as delaying disease progression | 222 cases | 24 weeks | Prospective, randomized, double-blind, placebo-controlled, multicenter study design | The special ginkgo biloba extract EGb 761 has definite clinical efficacy in outpatients with mild to moderate primary degenerative dementia of the Alzheimer type and multi-infarct dementia. It exerts therapeutic effects on both dementia subtypes, with a slightly more favorable efficacy trend observed in the dementia of the Alzheimer type subtype. | Markedly effective: 24 cases; effective: 16 cases; ineffective: 10 cases; total effective rate: 80%. | [S1] | See note 1 |
|  | Fufang Danshen Pian (with ingredients including *Salviae miltiorrhizae radix et rhizoma* and *Notoginseng radix et rhizoma*),  flavonoids, flavonols | Promoting blood circulation and removing blood stasis, which can dredge cerebral collaterals, improve cerebral blood supply, and relieve symptoms related to cognitive impairment | 128 cases | 36 weeks | Randomized controlled study design | It can effectively reduce the degree of blood stasis syndrome in vascular cognitive impairment (VCI) patients, with good safety; the incidence of adverse reactions has no significant difference from that of the control group, and no serious adverse events or abnormal liver and kidney functions have occurred. It can be used as an effective adjuvant drug for VCI treatment to delay disease progression. | The total effective rate of the control group is 70.3%, and that of the treatment group is 85.9%; the treatment group is significantly superior to the control group. | [153] | See Note 2 |
|  |  |  |  |  |  |  |  |  |  |
|  | Tianma Xingnao capsules (with ingredients including *Rehmanniae radix praeparata* and *Polygalae radix* ),  flavonols, flavonoids | Vascular mild cognitive impairment | 300 cases | 24 weeks | Randomized, double-blind, parallel-controlled, multicenter clinical study | Patients showed significant improvements in cognitive function and symptoms such as dizziness, tinnitus, and sleep disorders, which enhanced patients' quality of life. | It shifts the window period for dementia treatment to the cognitive impairment stage; while improving patients' cognitive function, it alleviates symptoms in multiple aspects, thereby achieving a comprehensive therapeutic effect. | [S2] | Clinical Trial Registry.gov:  ChiCTR2400084132 |
|  | Bushen Tongluo capsules (with ingredients including *Epimedii folium* and *Spatholobi caulis* )  flavonols, isoflavones | Advanced Parkinson's disease | 43 cases | 12 weeks | Randomized controlled design | Randomized controlled design the observation conclusions showed that compared with the control group, the treatment group had better improvements in the Webster clinical symptom score and a higher clinical effective rate. | The clinical effective rate of the treatment group was significantly higher than that of the control group; meanwhile, the improvement degree of the Webster clinical symptom score in the treatment group at week 12 was significantly better than that in the control group, which indirectly reflected that the proportion of effective cases in the treatment group was higher than that in the control group, and the combined treatment regimen had better efficacy. | [155] | See note 4 |
|  | [Qingkailing granule](https://pubmed.ncbi.nlm.nih.gov/38537336/),  (with ingredients including *Scutellariae radix* and *Lonicerae* *japonicae flos*),  flavonoids, flavonols | Encephalitis | 120 cases | 5-7 days | Randomized controlled design | The total effective rate of the observation group was 95.0%, which was significantly higher than that of the control group, and the difference in effective rate between the two groups was statistically significant. | Markedly effective: Body temperature returned to normal 2 days after treatment, and symptoms such as headache, irritability, and vomiting basically disappeared; effective: body temperature returned to normal 4 days after treatment, and the above-mentioned symptoms were significantly relieved; Ineffective: no improvement in symptoms after more than 5 days of medication. | [156] | See note 4 |
|  | Modified simiao wan (with ingredients including *Smilacis glabrae rhizoma* and *Achyranthis bidentatae radix*)  Flavonoids | Treating acute nonspecific myelitis | 37 cases | Not specified | Randomized controlled design | After treatment, most of the 37 patients showed significant improvements in muscle strength, sensation, and sphincter function, and complications were effectively controlled; cerebrospinal fluid and magnetic resonance reexaminations of typical cases returned to normal, achieving clinical cure; integrated traditional Chinese and Western medicine treatment can shorten the course of the disease, reduce sequelae, and its efficacy is superior to that of single traditional Chinese medicine or Western medicine treatment | Cured: 30 cases; improved: 6 cases; ineffective: 1 case; total effective rate: 97.3% | [157] |  |
| Phase Ⅰ | Quercetin,  flavonols | AD | 5 cases | 20-24 weeks | Self-controlled before and after treatment | In terms of safety, the treatment was well-tolerated with no premature drug withdrawal. | Only verified the safety and tolerability of the combined treatment with dasatinib and quercetin, as well as the central nervous system penetrability of dasatinib. | [158] | ClinicalTrials.gov:  [NCT04063124](http://clinicaltrials.gov/show/NCT04063124) |
|  |  | Early-stage AD | 5 cases | No more than 24 weeks | Randomized, double-blind, placebo-controlled | Patients with AD may have easier drug absorption due to early abnormalities of the blood-brain barrier, and intermittent drug administration may reduce adverse reactions; specific conclusions need to be verified after the completion of the trial. | By collecting data on cognition, function, neuroimaging and biomarkers, it provides a basis for subsequent Phase II trials, and the efficacy cannot be evaluated temporarily. | [S3] | ClinicalTrials.gov:  [NCT04063124](http://clinicaltrials.gov/show/NCT04063124) |
|  |  | Senolytic cell clearance | 12 cases | 3 weeks | Single-center, randomized, placebo-controlled pilot trial | Intermittent administration of dasatinib plus quercetin (D+Q) is feasible and generally well-tolerated, with no drug-related serious adverse events. Non-serious adverse events are more frequent in the D+Q group than in the placebo group, while no significant inter-group differences are observed in lung function and physical function. | Efficacy evaluation is not conducted due to small sample size and short observation duration. | [S4] | ClinicalTrials.gov:  NCT02874989 |
| Phase II | Anthocyanins | Inhibiting neuroinflammation, protecting nerve cells, delaying cognitive decline | 263  cases | 24 weeks | Parallel inter-group control and before-after self-control | The cognitive improvement effect of anthocyanins is "inflammation status-dependent": It is only effective in people at high risk of dementia with elevated inflammatory markers, and there is no benefit in people with low inflammation. | Markedly effective: 45 cases, accounting for 22.4%; effective: 0 cases, accounting for 0%; ineffective: 156 cases, accounting for 77.6% of the total population. | [S5] | ClinicalTrials.gov:  NCT02874989 |
|  |  | Delaying cognitive deterioration | 263 cases | 24 weeks | Randomized, double-blind, placebo-controlled clinical trial | The safety profile is favorable and tolerability is high; at 24 weeks, there is no significant inter-group difference in the primary outcome; there are no inter-group differences in secondary cognitive outcomes, and subgroups show a positive cognitive trend, though the interaction effect is not significant. | The trial confirms the safety and the trend of delayed cognitive deterioration, which requires verification through long-term studies with a larger sample size. | [159] | ClinicalTrials.gov:  NCT03419039 |
|  |  | Regulating ocular hemodynamics and assisting in slowing down visual field deterioration | 38 cases | 24 months | Randomized, placebo-controlled, Double-blind design | During the observation period, the serum endothelin (ET)-1 level in the breast cancer association consortium (BCAC) group increased gradually and reached the level of healthy people at 24 months; the serum ET-1 in the placebo group remained at a low level continuously; there were no significant changes in no-related indicators and antioxidant activity between the two groups. | BCACs can safely restore the abnormal serum ET-1 level, indirectly improve ocular blood flow, and assist in slowing down visual field deterioration, with no systemic or ocular side effects. | [S6] | ClinicalTrials.gov:  UMIN000004961 |
|  | Genistein,  isoflavones | Safety and preliminary efficacy in Patients with Prodromal AD | 52 cases | 12 months | Parallel control and before-after self-control | Genistein can inhibit the progression of amyloid deposition in the anterior cingulate cortex of patients, significantly improve their cognitive function in the Complutense Verba. | Markedly effective: 7 cases, accounting for 29.2% of the total completed cases; effective: 6 cases, accounting for 25.0% of the total completed cases; ineffective: 11 cases, accounting for 45.8% of the total completed cases; total effective rate: 13 cases, accounting for 54.2% of the total completed cases%. | [160] | ClinicalTrials.gov:  [NCT01982578](http://clinicaltrials.gov/show/NCT01982578) |
|  |  | Protecting neurological function and delaying cognitive decline | 32 cases | 12 months | Placebo-controlled, multi-center pilot clinical trial | Genistein can significantly delay the progression from prodromal AD to dementia in patients, and cognitive function in other cognitive tests shows a trend of improvement. | Markedly effective: 24 cases; effective: 16 cases; ineffective: 10 cases; total effective rate: 80%. | [S7] | ClinicalTrials.gov:  NCT01982578 |
|  | Quercetin,  flavonols | Improving cognitive function | 60 cases | 16 weeks | Pilot randomized, double-blind, placebo-controlled clinical trial | Enzogenol shows good safety and tolerability; there are no significant inter-group differences in working memory, episodic memory, post-concussion symptoms, or emotional scores, and dietary diversity and fat-free body mass have no significant impact on efficacy. | Markedly effective: 24 cases; effective: 16 cases; ineffective: 10 cases; total effective rate: 80%. | [S8] | Australian and New Zealand Clinical Trials Registry: ACTRN12610000107022 |
| Phase III | Epigallocatechin gallate (EGCG),  ‌flavanols | Delaying neurodegenerative progression associated with multiple system atrophy | 92 cases | 52 weeks | Prospective, randomized, double-blind, placebo-controlled parallel group | Final efficacy data have not yet been published; only the feasibility of recruitment has been confirmed. focus should be placed on liver function monitoring. The study design has initially shown feasibility, and efficacy data are expected to be obtained in 2017. | It exhibits potential neuroprotective effects manifested by a significant reduction in striatal volume loss on magnetic resonance imaging. | [S9] | ClinicalTrials.gov:  NCT03608091 |
|  | Genistein,  isoflavones | For attention deficit hyperactivity disorder (ADHD) and attention deficit disorder in children, exerting potential therapeutic and improvement effects through multiple pathways | 144 cases | 10 weeks | Double control method | No actual results have been conducted or published yet; only the research design objectives are clarified, which is to verify the improvement effect of pycnogenol on ADHD behaviors as well as oxidative, immune, and neurochemical indicators, and there are no observation conclusions available for the time being. | The core for judging efficacy lies in the core pathological characteristics of ADHD, while the secondary indicators are used to supplement the evaluation of comprehensiveness of efficacy and safety. | [S10] | ClinicalTrials.gov:  [NCT02700685](http://clinicaltrials.gov/show/NCT02700685) |
|  | Ginkgo biloba leaf extract 761,  flavone glycosides | Improving cognitive and social functions through antioxidant effects, cell membrane protection, and neurotransmission regulation | 309 cases | 52 weeks | Multicenter, randomized, double-blind, placebo-controlled, parallel-group study | It is safe and can stabilize or even improve patients' cognitive and social functions for 6-12 months; there is no significant difference in clinical global impression of change, and the incidence of adverse events is similar to that in the placebo group. | Efficacy evaluation shows that in the intention-to-treat analysis, the EGb group has better scores in the Alzheimer's disease assessment scale-cognitive subscale (ADAS-Cog) and Geriatric rating scale for cognitive impairment (GERRI) than the placebo group; 27% of patients in the EGb group achieve an improvement of ≥4 points in ADAS-Cog, 37% show improvement in GERRI, and the efficacy is more significant in the AD subgroup. | [S11] | See Note 1 |
| Phase IV | Sanqi Tongshu capsule (with the ingredient: *Notoginseng radix et rhizoma*),  flavonols | Ischemic cerebrovascular disease | 48 cases | 28 days | Open-label clinical trial | After 28 days of treatment, patients' neurological deficit scores and Barthel index scores were significantly improved compared with those before treatment. It is safe and effective in the treatment of ischemic cerebrovascular disease, and can improve neurological function and activities of daily living. | All scores were significantly increased after treatment. | [154] |  |

**Note**: 1. References [S1,154,S11]: The studies were conducted in 1997, which was earlier than the popularization stage of the mandatory international clinical trial registration system. At that time, the International Committee of Medical Journal Editors had not yet introduced the mandatory requirement that "clinical trials must be registered before publication" (this requirement was gradually promoted after 2005), and the global coverage of international registration platforms such as ClinicalTrials.gov was relatively low. Early studies on verifying the efficacy of already marketed extracts were generally not included in the scope of public registration, so no identification code was assigned.

1. Reference [153]: This study is a "clinical observational study on an already marketed traditional Chinese medicine". Although it adopted a randomized controlled design, it was not a "new drug registration clinical trial". In 2016, Good Clinical Practice for Drugs did not include such "efficacy observation of adjuvant therapy" in the scope of mandatory registration; the researchers had insufficient awareness of registration and only published the study results through journals without disclosing registration information on platforms such as the ChiCTR. Therefore, no identification code was assigned.

3. Reference [155]: This study is an "observation on the efficacy of combined use of an already marketed traditional Chinese medicine and Western medicine", which belongs to the "re-evaluation of clinical application of already marketed drugs" rather than a new drug clinical trial. In 2014, China's registration requirements for "studies on combined use of already marketed traditional Chinese medicines" had not been strictly implemented. The study only passed ethical review within the institution and was not registered on public platforms such as ChiCTR, so no identification code was assigned.

4. References [156,157]: According to the document notes, the core reason is that the studies were conducted earlier than the establishment of China's registration system: the studies were carried out in 2000, while the Chinese ChiCTR was established in 2007. At that time, there was no unified public registration platform for clinical trials in China; the 2003 version of GCP for Drugs only targeted "new drug clinical trials" and had no mandatory registration requirements for "observational studies on the treatment of infectious diseases with already marketed traditional Chinese medicines". The researchers had insufficient awareness of "public registration of clinical trials" and only published the results through journals, so no identification code was assigned.

### Table S4. Clinical data on nanocarriers for overcoming the BBB

| **ClinicalTrials.gov ID** | **Nanocarrier types** | **Phase/Study Type** | **Statuses** |
| --- | --- | --- | --- |
| NCT01960348 | lipid nanoparticle | 3 | Completed |
| NCT03993171 | Gold nanocrystals | 2 | Active, not recruiting |
| NCT03815916 | Gold Nanocrystals | 2 | Completed, [with results](https://clinicaltrials.gov/study/NCT03815916?term=NCT03815916&rank=1&tab=results) |
| NCT04380194 | nano-composite | Observational | Completed |
| NCT00734682 | Nanoliposomal CPT-11 | 2 | Completed |
| NCT04264143 | HPBCD | 1 | Completed |
| NCT05212311 | Chitosan | Not Applicable | Completed |
| NCT01246336 | Carbon nanotubes and gold nanoparticles | Observational | Completed |
| NCT00734682 | Nanoliposomal | 1 | Completed |
| NCT00313599 | albumin-stabilized nanoparticle | 1 | Completed |
| NCT03736720 | liposomal | 2 | Terminated, [with results](https://clinicaltrials.gov/study/NCT03815916?term=NCT03815916&rank=1&tab=results) |
| NCT05040373 | patisiran-LNP | Observational | Recruiting |
| NCT06572475 | USPIO or ultrasmall superparamagnetic iron oxide nanoparticles | Not Applicable | Recruiting |
| NCT07064083 | Intracanal nano-ketrolac | Early Phase 1 | Recruiting, new |
| NCT06855368 | biomimetic anti-inflammatory nanoparticles | Observational | Recruiting |
| NCT01125215 | 0.75% capsaicin nanoparticle cream | 2, 3 | Unknown status |
| NCT03747328 | Nab-sirolimus | 2 | Withdrawn |
| NCT03843710 | Gold Nanocrystals | 2 | Withdrawn |
| NCT04480333 | / | 1 | Unknown status |
| NCT04601051 | Lipid nanoparticles | 1 | Active, not recruiting |
| NCT02022644 | nanoliposomal | 1 | Completed |
| NCT03086616 | Nanoliposomal | 1 | Completed |
| NCT06491992 | Chitosan | Not Applicable | Recruiting |
| NCT02459015 | Chitosan | Not Applicable | Terminated |

### Table S5. Clinical data on the use of ultrasound to overcome the BBB

| **ClinicalTrials.gov ID** | **Phase/Study Type** | **Statuses** |
| --- | --- | --- |
| NCT03119961 | 1, 2 | Completed |
| NCT03744026 | 1, 2 | Completed |
| NCT02253212 | 1, 2 | Completed |
| NCT01240356 | 1, 2 | Completed, [WITH RESULTS](https://clinicaltrials.gov/study/NCT01240356?term=NCT01240356&rank=1&tab=results) |
| NCT03296852 | Early Phase 1 | Completed |
| NCT04804709 | 1 | Terminated, [WITH RESULTS](https://clinicaltrials.gov/study/NCT04804709?term=NCT04804709&rank=1&tab=results) |
| NCT04091503 | 1 | Completed |
| NCT02986932 | Not Applicable | Completed |
| NCT03616860 | Not Applicable | Completed |
| NCT03626896 | Not Applicable | Completed |
| NCT03551249 | Not Applicable | Completed |
| NCT04118764 | Not Applicable | Completed, [WITH RESULTS](https://clinicaltrials.gov/study/NCT04118764?term=NCT04118764&rank=1&tab=results) |
| NCT03712293 | Not Applicable | Completed |
| NCT04446416 | Not Applicable | Completed |
| NCT05383872 | Not Applicable | Completed |
| NCT06214624 | Not Applicable | Completed |
| NCT05902169 | 3 | Recruiting |
| NCT06496971 | 3 | Recruiting |
| NCT05879120 | 2 | Withdrawn |
| NCT05864534 | 2 | Recruiting |
| NCT04528680 | 1, 2 | Active, not recruiting |
| NCT04417088 | 1, 2 | Active, not recruiting |
| NCT04440358 | 1, 2 | Active, not recruiting |
| NCT05615623 | 1, 2 | Recruiting |
| NCT06329570 | 1, 2 | Not yet recruiting |
| NCT05630209 | 1, 2 | Recruiting |
| NCT07179328 | 1 | Recruiting, New |
| NCT05293197 | 1 | Recruiting |
| NCT06600880 | 1 | Not yet recruiting |
| NCT07193953 | 1 | Recruiting |
| NCT06585384 | 1 | Not yet recruiting |
| NCT05762419 | 1 | Recruiting |
| NCT05469009 | Early phase 1 | Active, not recruiting |
| NCT03671889 | Not Applicable | Recruiting |
| NCT06474013 | Not Applicable | Not yet recruiting |
| NCT05565443 | Not Applicable | Recruiting |
| NCT02343991 | Not Applicable | Active, not recruiting |
| NCT06158789 | Not Applicable | Active, not recruiting |
| NCT04063514 | Not Applicable | Enrolling by invitation |
| NCT05733312 | Not Applicable | Recruiting |
| NCT06522295 | Observational [Patient Registry] | Not yet recruiting |
| NCT05273216 | Observational | Recruiting |
| NCT05755399 | Not Applicable | Recruiting |
| NCT04614493 | 2 | Unknown status |
| NCT04021420 | 1, 2 | Unknown status |
| NCT03739905 | Not Applicable | Unknown status |
| NCT04526262 | Not Applicable | Unknown status |
| NCT04998864 | Observational | Unknown status |
| NCT04370665 | Not Applicable | Unknown status |
| NCT03608553 | Not Applicable | Unknown status |
| NCT03321487 | Not Applicable | Unknown status |
| NCT03714243 | Not Applicable | Unknown status |
| NCT03709290 | Observational | Unknown status |
| NCT01555684 | Not Applicable | Withdrawn |
| NCT04877184 | Not Applicable | Unknown status |
| NCT04667715 | Not Applicable | Withdrawn |

# Supplementary Figure

**Figure S1.** Roadmap for *Challenges and prospects*

**
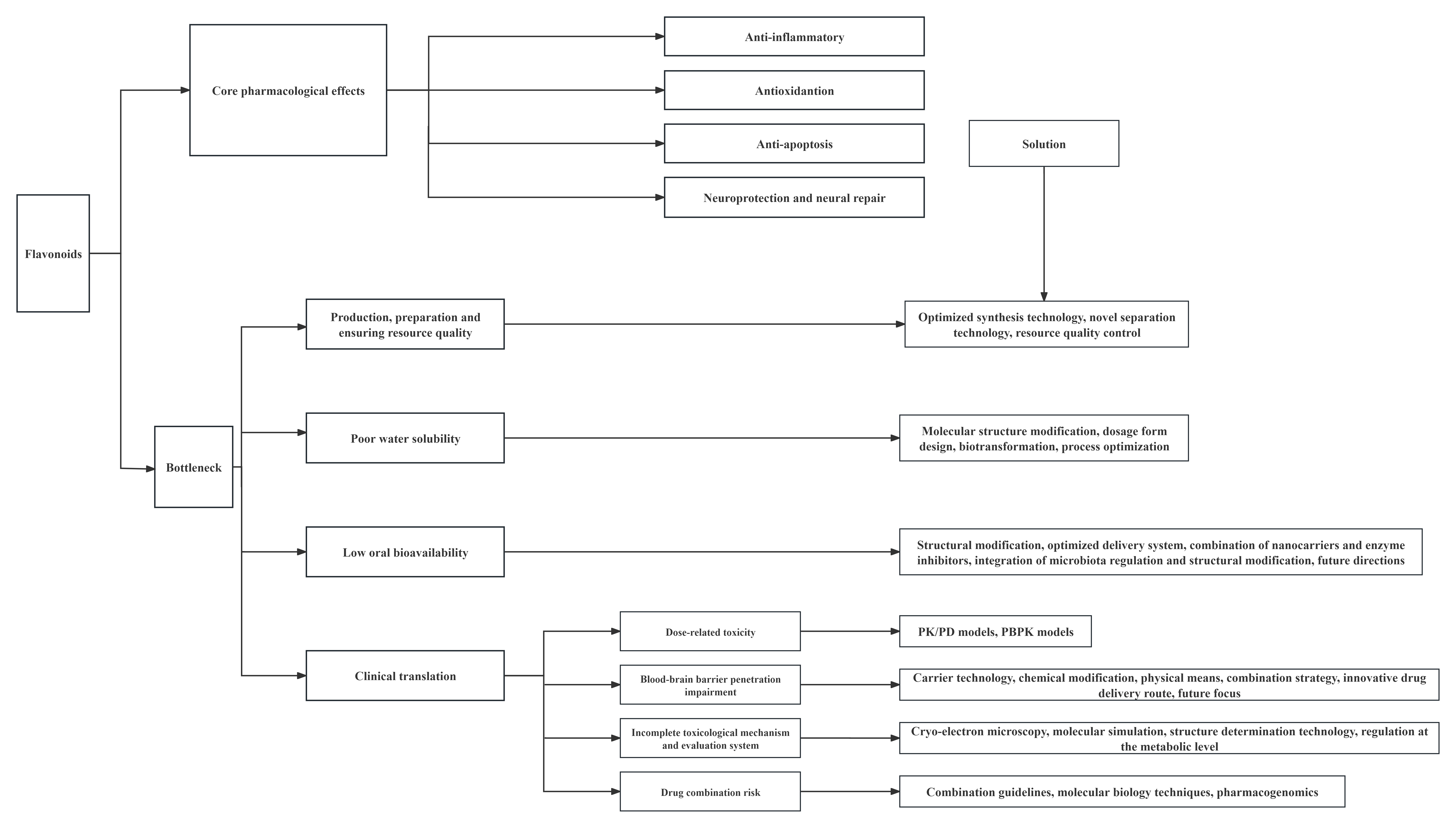
**

**References:**

1. Kanowski S, Herrmann WM, Stephan K, Wierich W, Hörr R. Proof of efficacy of the Ginkgo biloba special extract EGb 761 in outpatients suffering from mild to moderate primary degenerative dementia of the Alzheimer type or multi-infarct dementia. Phytomedicine. (1997) 4(1):3-13. doi: 10.1016/S0944-7113(97)80021-9
2. China Net. The treatment window for dementia has been moved forward to the cognitive impairment stage, according to professor Lin Yaming's latest clinical research progress on Tianma Xingnao capsules. (2024). https://life.china.com/2024-09/02/content_353986.html.(Accessed September 28, 2025)
3. Gonzales MM, Garbarino VR, Marques Zilli E, Petersen RC, Kirkland JL, Tchkonia T, et al. Senolytic therapy to modulate the progression of Alzheimer's disease (SToMP-AD): a pilot clinical trial. J Prev Alzheimers Dis. (2022) 9(1):22-29. doi: 10.14283/jpad.2021.62
4. Borda MG, Barreto GE, Baldera JP, de Lucia C, Khalifa K, Bergland AK, et al. A randomized, placebo-controlled trial of purified anthocyanins on cognitive function in individuals at elevated risk for dementia: analysis of inflammatory biomarkers toward personalized interventions. Exp Gerontol. (2024) 196:112569. doi: 10.1016/j.exger.2024.112569
5. Aarsland D, Khalifa K, Bergland AK, Soennesyn H, Oppedal K, Holteng LBA, et al. A randomised placebo-controlled study of purified anthocyanins on cognition in individuals at increased risk for dementia. Am J Geriatr Psychiatry. (2023) 31(2):141-151. doi: 10.1016/j.jagp.2022.10.002
6. Yoshida K, Ohguro I, Ohguro H. Black currant anthocyanins normalized abnormal levels of serum concentrations of endothelin-1 in patients with glaucoma. J Ocul Pharmacol Ther. (2013) 29(5):480-487. doi: 10.1089/jop.2012.0198
7. Viña J, Borrás C, Mas-Bargues C. Genistein, a Phytoestrogen, delays the transition to dementia in prodromal Alzheimer's disease patients. J Alzheimers Dis. (2024) 101(s1):S275-S283. doi: 10.3233/JAD-240308
8. Theadom A, Mahon S, Barker-Collo S, McPherson K, Rush E, Vandal AC, et al. Enzogenol for cognitive functioning in traumatic brain injury: a pilot placebo-controlled RCT. Eur J Neurol. (2013) 20(8):1135-1144. doi: 10.1111/ene.12099
9. Levin J, Maaß S, Schuberth M, Respondek G, Paul F, Mansmann U, et al. The PROMESA-protocol: progression rate of multiple system atrophy under EGCG supplementation as anti-aggregation-approach. J Neural Transm (Vienna). (2016) 123(4):439-445. doi: 10.1007/s00702-016-1507-8
10. Verlaet AA, Ceulemans B, Verhelst H, Van West D, De Bruyne T, Pieters L, et al. Effect of pycnogenol® on attention-deficit hyperactivity disorder (ADHD): study protocol for a randomised controlled trial. Trials. (2017) 18(1):145. doi: 10.1186/s13063-017-1879-6
11. Le Bars PL, Katz MM, Berman N, Itil TM, Freedman AM, Schatzberg AF. A placebo-controlled, double-blind, randomized trial of an extract of Ginkgo biloba for dementia. North American EGb study group. JAMA. (1997) 278(16):1327-1332. doi: 10.1001/jama.278.16.1327
